# Microemulsion Microstructure(s): A Tutorial Review

**DOI:** 10.3390/nano10091657

**Published:** 2020-08-24

**Authors:** Giuseppe Tartaro, Helena Mateos, Davide Schirone, Ruggero Angelico, Gerardo Palazzo

**Affiliations:** 1 Department of Chemistry, and CSGI (Center for Colloid and Surface Science), University of Bari, via Orabona 4, 70125 Bari, Italy; giuseppe.tartaro@uniba.it (G.T.); helenamateos.cuadrado@uniba.it (H.M.); davide.schirone@uniba.it (D.S.); 2 Department of Agricultural, Environmental and Food Sciences (DIAAA), University of Molise, I-86100 Campobasso, Italy; angelico@unimol.it

**Keywords:** microemulsions, wormlike micelles, packing parameter, flexible surface model, hydrophilic–lipophilic difference (HLD), net average curvature (NAC)

## Abstract

Microemulsions are thermodynamically stable, transparent, isotropic single-phase mixtures of two immiscible liquids stabilized by surfactants (and possibly other compounds). The assortment of very different microstructures behind such a univocal macroscopic definition is presented together with the experimental approaches to their determination. This tutorial review includes a necessary overview of the microemulsion phase behavior including the effect of temperature and salinity and of the features of living polymerlike micelles and living networks. Once these key learning points have been acquired, the different theoretical models proposed to rationalize the microemulsion microstructures are reviewed. The focus is on the use of these models as a rationale for the formulation of microemulsions with suitable features. Finally, current achievements and challenges of the use of microemulsions are reviewed.

## 1. Introduction

As a matter of fact, water and oil do not mix. There are several applications in which this is a bitter truth and the coexistence of oil and water as separate macroscopic phases, interacting just through a small interface, is highly disappointing.

Of course, one can stir the mixture to break the two phases in droplets and increase the interfacial area but, without additional tricks, such an approach is destined to fail due to the conspiracy of two factors. On the one hand, the water/oil interfacial tension opposes the increase in area. On the other hand, the fate of these droplets is a fast and irreversible coalescence so that the system goes back to a macroscopic two-phase system. The reformation of the macroscopic two-phase system is driven by van der Waals attraction between the droplets of the same material. Therefore, when dealing only with oil and water, there is nothing that can oppose the droplets contact and subsequent coalescence.

To improve the stability of the dispersion, one must add some chemicals that sit at the interface and prevent collision and coalescence. The requirement of a strong affinity for the interface is thermodynamically translated into the ability to reduce the interfacial tension through the Gibbs adsorption Equation [1].

Upon the addition of suitable surfactants, one can provide kinetic stability to water and oil dispersions in the form of macroemulsion or simply emulsion droplets. From a thermodynamic point of view, the emulsion is still a nonequilibrium state but the presence of emulsifier (surfactant) at the interface between water and oil imparts repulsive interactions that, at least in part, counteract the van der Waals attraction so that the lifetime of the emulsion can be considerably long. The emulsion droplets are spherical to minimize the surface-to-volume ratio, but their size remains in the µm range so that the interface is essentially flat at the molecular length scale. Micron sizes imply the emulsion is milky and quite viscous. Being thermodynamically unstable, a considerable amount of energy is required to form emulsions and care in the formulation is necessary to preserve their structure. Visually, emulsions are opaque milky systems that can be very viscous for large enough volume fraction of the dispersed phase. A representative and tasty example is the well-known mayonnaise sauce.

However, if the choice of surfactant is appropriate and its concentration is high enough, a totally different outcome can be observed: the system made of oil, water, and surfactant (s) becomes optically transparent and thermodynamically stable and this is what we call a microemulsion (µE). In 1959, when Schulman proposed to call “microemulsion” [2] the optically isotropic and transparent, thermodynamically stable mixtures of oil, water, and surfactants, the prefix “micro” was used in the sense of “very-small” without any link to the actual length scale. The optical transparency of the µE implies that their microstructure must be characterized by length scales that are submicrometric (usually below 100 nm). Such low sizes are associated with a huge interfacial area that can be attained without any energy input only because the interfacial tension (*γ*) is very low (in many cases *γ* was found to be as low as 10^−4^ mJ/m^2^). For ultralow interfacial tension, the constraint of the spherical shape, as the one that assures the lowest surface-to-volume ratio, becomes insignificant and for many µEs, the oil or aqueous domains are arranged in shapes very different from globular. The name microemulsion is misleading since they are nanosized and often not formed by droplets.

To further increase the terminology complexity, nowadays the thermodynamically unstable emulsion formed by droplets with sizes ~200 nm is called nanoemulsion.

However, differences between emulsion and µE can be told at a glance since, usually, µEs have the transparency and low viscosity of water. When the amount of water and oil solubilized is very high, they can have a bluish appearance.

It should be stressed that the µE formation takes place in a “sweet-spot” of composition that often requires more than one kind of surfactant and depends on the composition of the oil-phase, on the salinity of the water phase and the temperature. Very often further components such as medium-chain alcohols are required so that the formulation of a µE is not an easy task. For this reason, the former applications of µEs were limited to a few products designed by trial-and-error approaches (carnauba wax polish, cutting oil, pine oil disinfectant, and a few others) [3]. Things changed drastically in the occasion of the oil crisis in the seventies, and the associated one-order of magnitude increase in the petroleum price. The ultralow interfacial tension attained in certain µE was exploited to detach the oil secluded in the rock pores. In addition, the conditions of ultralow γ are the ones at which the µE dissolves the largest amount of oil. As a bonus, under these conditions of ultralow interfacial tension, the excess oil (not dissolved in the µE) is easily emulsified in form of oil droplets by the flow of surfactant solution pumped in the reservoir but, at rest, the oil separates nicely. Sounds too good to be true! The real problem is finding the right mix of surfactants and additives (ideally the cheapest ones) that, added to the available water (often very salty) forms the desired µE with that specific crude oil. This is not a goal that can be achieved through a blind random walk among the millions of possible formulations. Instead, it requires a deep understanding of the physical chemistry of the µE systems starting from the experimental determination of their microstructure and how it impacts the interfacial tension. Different experimental methods, such as small-angle scattering from X-ray (SAXS) and neutrons (SANS), diffusion NMR, and Cryogenic Electron Microscopy (cryoTEM and cryoSEM) have contributed to the elucidation of the different microstructures that can be found on these fascinating mixtures. The µEs microstructure can be idealized as a set of interfaces dividing polar and apolar domains. Such an interfacial film can arrange itself in a variety of shapes depending on the intensive variables of the system. The crucial role of the interfacial film’s curvature is at the basis of all the theories about the µE thermodynamics. Elegant and sophisticated theoretical models have been developed to rationalize several aspects of µEs and, recently, semiempirical models have become part of the toolbox of µE formulation.

Within the last five decades, µEs have also become increasingly significant in the industry. Besides their application in enhanced oil recovery, they are widely used in many other applications. However, most of the latest reviews on the models that describe µEs structure and behavior go back to the 1990s. Dealing with such a diverse span of applications is no surprise that the reviews that have come out over the last 20 years, like the synthesis of organic nanoparticles [4] or the use of Ionic Liquids (ILs) [5], deal with very specific application areas of µEs. On the other hand, in the last 10 years, there is a lack of papers dedicated to summarizing the knowledge we have gained on the microstructure of µEs and to critically compare the different models proposed to describe and formulate these systems.

This tutorial review is addressed to scientists, lacking previous experience of μEs, that are interested in their potential use in their own research in cutting edge applications (drug delivery, synthesis of composite (nano)materials, as chemical reaction media, etc.).

The review starts with an overview of what microstructures a µE can have and on the techniques of choice to test it including the study of their phase behavior. Then we review the main theoretical models based on the effective packing parameter of the surfactant, on spontaneous curvature of the flexible interface, and the more recent hydrophilic–lipophilic difference (HLD) in combination with the net average curvature (NAC) estimation.

The last section deals with the achievements of µE applications and future challenges in their formulation.

## 2. Microemulsion Structures

### 2.1. Shape and Size of Self-Assembled Surfactant Structures

**Figure 1:**
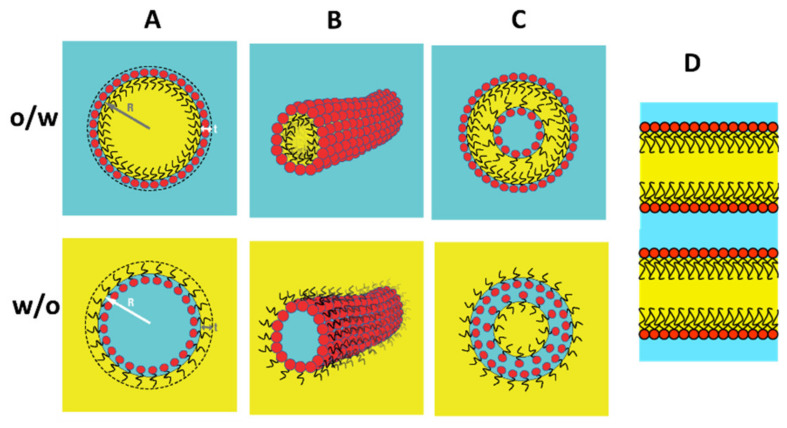
Cartoon of self-assembled surfactant structures in microemulsions. Upper row oil-in-water, lower row water-in-oil. (**A**) Spherical micelles; (**B**) cylindrical micelles; (**C**) vesicles; (**D**) bicontinuous planar interfaces.

µEs are thermodynamically stable, homogenous mixtures of water and another otherwise immiscible liquid (hereafter oil) based on surfactant self-assemblies and as such they share several features with classical surfactant phases such as micellar solutions and lyotropic liquid crystalline phases (this review does not cover the topic of the “surfactant-free” μEs [6]). In binary surfactant–water systems, it is possible to encounter spherical and cylindrical micelles [7,8], and one can imagine to incorporate oil in the apolar region of these micelles obtaining the equivalent oil-in-water (o/w) µEs (Figure 1A,B, o/w row). Symmetrically, one can imagine to dissolve the surfactant in the oil and add small amounts of water to these systems obtaining water pools (spherical or cylindrical) separated from the oil by a surfactant monolayer, a situation that is called a water-in-oil (w/o) µE (Figure 1A,B, w/o row). When only a small amount of water is dissolved these entities are called reverse micelles and the specular o/w situation is referred to as (swollen) direct micelles.

Direct and reverse micelles behave as their counterparts in the surfactant/water binary systems [9]. Spherical micelles tend to be monodispersed in size with a radius *R* that depends only on the ratio between the volume of the dispersed phase and the total interfacial surface. For a w/o spherical reverse micellar solution made by *n_w_* molecules of water (volume fraction *ϕ_w_*) and *n_s_* molecules of surfactant (volume fraction *ϕ_s_*) with a polar-headgroup area α and a molecular volume *v_s_*, we have

(1) $$R=\frac{3v_{w}}{\alpha }\frac{n_{w}}{n_{s}}=\frac{3\phi_{w}l_{s}}{\phi_{s}}.$$

where *v_w_* is the water molecular volume (30 Å^3^) and the second equality is written in terms of an effective surfactant length $l_{s}\equiv \frac{v_{s}}{\alpha }$. The same holds for spherical o/w µE simply by exchanging the water with the oil. Is worth emphasizing that *R* in Equation (1) is the radius of the spherical surface enveloping the dispersed volume (see Figure 1A).

For cylindrical micelles, the free energy penalty associated with the two ends is the driving force for micellar elongation so that they easily elongate, to an extent, such that they behave as polymer solutions (polymerlike or wormlike micelles), giving the system viscoelastic properties [10].

**Figure 2:**
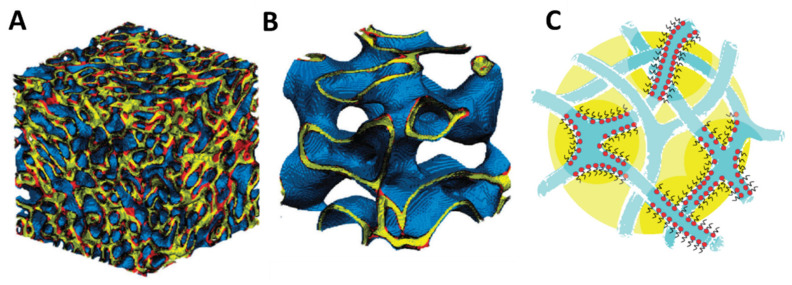
(**A**,**B**) Simulation of bicontinuous microemulsions (µE) with different compositions and rigidity. The side lengths of figures are 63 nm. For the illustrations, water is blue, oil is yellow, and the surfactant is red (only separation surfaces between the domains are represented. (**A**) Oil-rich (57% oil; 26% surfactant) flexible interface; (**B**) water-rich (21% oil, 15% surfactant) ultrarigid locally lamellar. Adapted from [13] with permission from the PCCP Owner Societies. (**C**) Pictorial description of a dilute, highly asymmetric bicontinuous water-in-oil (w/o) µE.

Both o/w and w/o µEs can be described as collections of (oil or water) discrete domains dispersed in a continuous solvent (water or oil). Surfactants in water also form structures based on (locally) flat bilayers such as vesicles and liquid crystalline lamellar phases or the weird cubic bicontinuous phases [1,11]. If one imagines the surfactant tail region to swell with oil, the bilayer is transformed into two monolayers separating oil and water domains (see Figure 1D). Accordingly, one can encounter direct and reverse vesicles [12] (Figure 1C) and lamellar phases that can swell both in water and oil domains (Figure 1D). Furthermore, the transformation of the surfactant bilayer into two monolayers separated by a liquid oil layer reduces sensibly the intrinsic rigidity of the bilayer. The entropy associated with thermal fluctuations of the locally flat surfactant film very often drives the system out from the liquid crystal realm into structures that are isotropic liquid with intertwined continuous domains of water and oil separated by surfactant monolayers. These are collectively called bicontinuous µEs and, since bicontinuity can only occur in a three-dimensional space, their structures are difficult to grasp in two dimensions (two examples of these microstructures, differing in composition and film rigidity, are shown in Figure 2).

The degree of flexibility of the surfactant film in bicontinuous µEs is described by a persistence length *ξ* (the interface is essentially flat at scales smaller than *ξ*). Theoretical treatment indicates that the persistence length is extremely sensitive to the rigidity of the film (quantified by a curvature modulus *κ_c_*) according to [14]:

(2) $$\xi =l_{s}\times exp\left(\frac{2\pi \kappa_{c}}{k_{b}T}\right).$$

On the other hand, a bicontinuous µE can be thought of as a distribution of domains of average size *ξ* randomly filled by water and oil. It was demonstrated that such a domain size must fulfill the following relation among the water and oil volumes and the available interfacial area density (specific area) $\sum =\frac{\phi_{s}}{l_{s}}=\frac{n_{s}\alpha }{V}$ [14,15]:

(3) $$\xi =\frac{6\phi_{w}\phi_{o}}{\sum }=\frac{6\phi_{w}\phi_{o}}{n_{s}\alpha }V=\frac{6\phi_{w}\phi_{o}}{\phi_{s}}l_{s}.$$

Further details on the fascinating microstructure(s) of bicontinuous µEs will be given in Section 3 below. Here, it is worth emphasizing that, geometrically, these systems have a huge interfacial area compared with disconnected (o/w and w/o) µEs. This is because the compositions at which bicontinuous µEs exist are characterized by ultralow interfacial tensions with both water and oil. As a matter of fact, the lowest interfacial tension corresponds to equal volumes of solubilized water and oil. At this point, the µEs are said to be “balanced” and the corresponding compositions are referred to as “optimal composition” because it is the best formulation in the applications where the goal is to achieve large solubilization (as in enhanced oil recovery). It should be said that, for some systems, a form of bicontinuity is found at very low amounts of dispersed phase [16,17,18]. The basic micellar structure here is the cylindrical one and the branching of these is allowed thus forming asymmetric bicontinuous networks [19] like the one sketched in Figure 2C that are often filed as branched wormlike micelles [18] and are reviewed in some details in Section 4.

### 2.2. Experimental Determination of the Microstructure

#### 2.2.1. Diffusion NMR

From the above brief excursus about the microstructures, a first strong peculiarity of µEs emerges. Which component is playing the role of the continuous phase cannot be taken for granted and, instead, must be checked experimentally. The straightforward approach to the connectivity determination that answers one single sample is an NMR technique based on the use of pulsed magnetic gradients designed by several acronyms (PGSE-NMR, PFG-NMR, DOSY) that, in recent years, has taken hold the name diffusion NMR (dNMR).

dNMR allows the determination of the self-diffusion coefficients of different chemical species (identified by their NMR signal). This is a powerful tool to discriminate the connectivity of the µEs [20,21]. In the case of an o/w µE, surfactant and oil share the same self-diffusion coefficients (*D_s_* ≈ *D_oil_* *D_water_*), while in the case of a w/o µE *D_water_* ≈ *D_s_* *D_oil_*. Finally, in the case of bicontinuous systems, the diffusion coefficients of the three components are uncorrelated, but water and oil have diffusion coefficients close to those of pure components and usually much higher than that of the surfactant’s self-diffusion (*D_water_* ≈ *D_oil_* *D_s_*).

Further insight can be gained if the measurements are taken at different compositions along a properly chosen dilution path.

In the case of disconnected domains (o/w or w/o µEs), the self-diffusion coefficient of the dispersed phase is related to the hydrodynamic radius (*R_h_*) by the Stokes–Einstein equation:

(4) $$D{}^{\circ}=\frac{k_{b}T}{6\pi \eta R_{h}}$$

where *D*° is the diffusion coefficient at infinite dilution. If the viscosity of the continuous phase *η* is expressed as mPa s at 25 °C Equation (2) is $D{}^{\circ}=\frac{21}{R_{h}\left(Å\right)}$, where *D*° is in units of 10^−10^ m^2^ s^−1^.

Strictly what is measured by dNMR is the observed diffusion coefficient (*D*) that is related to the diffusion coefficient at infinite dilution by the virial equation:

(5) $$D=D{}^{\circ}\left(1+k_{NMR}\phi \right)$$

where *ϕ* is the volume fraction of the dispersed phase and *k*_NMR_ is a constant depending on the inter droplets interactions. For a relatively dilute ensemble of particles (*ϕ* small) *D*~*D°.* However, if an accurate sizing is required, the effect of interparticle interaction and obstruction must be considered (more details are given in the section on light-scattering further on).

For spherical particles, the hydrodynamic radius depends on the radius of the dispersed phase domain (*R* in Equation (1)) according to *R_h_ = t + R,* where *t* represents either the length of apolar surfactant tail *l_s_* for w/o µE or the thickness of (hydrated) polar headgroup for o/w µE (see Figure 1).

Upon loading the system with the dispersed phase, one can check if the linearity of Equation (1) holds (i.e., there are spherical droplets), and in that case, it is possible to estimate *α* and *l_s_* [22].

An analogous approach can be undertaken in the case of bicontinuous µEs, for which characteristic dependences, on the surfactant volume fraction, of the diffusion coefficient of surfactant (*D_s_* = *D*_0,*s*_ [2/3 − *µϕ_s_^2^*], where *D*_0,*s*_ is the lateral self-diffusion coefficient of the surfactant), and of the molecules diffusing in the continuous domains (*D_oil_* or *D_w_* = *D*_0_ [2/3 − *µϕ_s_*], where *D*_0_ is the diffusivity of the neat continuous phase considered) have been proposed theoretically and confirmed experimentally (*µ* is a constant that depends on the topology of the dispersed phase) [23,24,25].

Finally, it is worth stressing how different chemical shifts that label different species allows simultaneous measurements of their individual dynamics. In complex formulations (e.g., many surfactants, cosurfactants, and additives), this potentially permits the quantification of multiple associated species and partition equilibria [21].

#### 2.2.2. Electrical Conductivity

**Figure 3:**
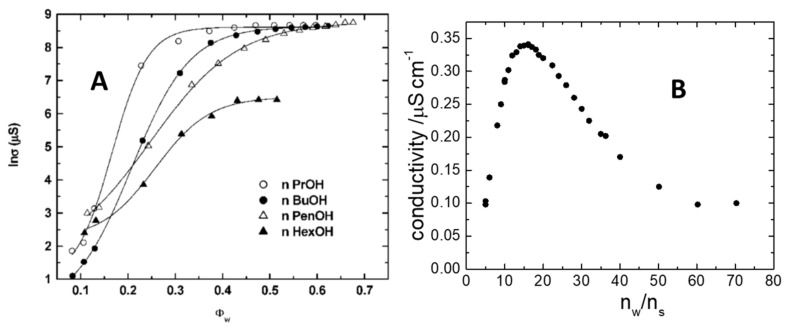
(**A**) Dependence of the logarithm of conductivity on the water volume fraction for the w/o µE water/SDS + Myrj 45/cyclohexane in the presence of different alcohols at 30 °C. Reprinted with permission from [26]. Copyright (2004) American Chemical Society. (**B**) Conductivity as a function of the water/surfactant molar ratio for the w/o µE water/CTAB/pentanol/hexane. Adapted with permission from [22]. Copyright (1996) American Chemical Society.

Conductivity (*χ*) is an inexpensive technique that can be very useful in the characterization of µEs (provided the formulation contains salt or/and ionic surfactant). At the simplest level of application, it allows discriminating between o/w (that has a conductivity of the order of mS/cm) and w/o µE (with a conductivity of the order of µS/cm). The conductivity of bicontinuous µEs is between these values and depends dramatically on the degree of connectivity of the aqueous channels. Accordingly, it is quite easy to follow the transition from w/o to bicontinuous µE or from o/w to bicontinuous µE upon tuning an intensive parameter such as temperature or composition. An example is shown in Figure 3A, where the water-induced percolation of a complex five-component µE made of water, two surfactants (SDS + Myrj45), alcohol, and cyclohexane, has been studied in detail by changing the alcohol used as cosurfactants [26]. The steep rise in conductivity (six orders of magnitude) upon a moderate increase in water content is typical of the w/o → bicontinuous → o/w transition reflecting the percolation of aqueous domains. Note that, sometimes, one observes an opposite behavior. In reverse asymmetric bicontinuous networks, like the ones sketched in Figure 2C, dilution with water causes a dramatic decrease in conductivity [16]. Such an antipercolative behavior is indicative of a drastic modification of the microstructure that changes from interconnected domains to closed spherical reverse micelles.

Although the w/o µEs are often described as nonconductive they show a nonzero conductivity of few µS/cm or less. These are values that can be measured with an accurate conductometer. In Figure 3B, the typical trend of the conductivity for spherical reverse micelles is shown along a water dilution line (i.e., loading with water a µE with a constant amount of surfactant and oil). It is clear the conductivity presents a well-defined maximum when plotted against the molar ratio water/surfactant that, according to Equation (1), is proportional to the water pool radius of the w/o µE.

Among ternary systems without cosurfactants, we can mention the AOT-based w/o µEs for which most of the conductivity curves exhibit bell-shaped trends similar to those reported in Figure 3B [27]. Such behavior was rationalized based on the charge fluctuation model, considering the Born energy needed to charge a sphere in a dielectric medium. The larger the micelle is, the higher is the excess charge sitting on it [28]. However, larger size implies also a smaller diffusion of the charge bearing micelles because of the increase of the hydrodynamic size (Equation (1) [29]. It should be noted that the electric charge is forced to reside on the surface of the polar domain (the aqueous pool) while hydrodynamically what matters is the overall reverse micellar size including the apolar surfactant tails (and possibly some solvation oil molecule) as shown in the w/o case of Figure 1A [22]. The mismatch between hydrodynamic radius *R_h_* and the water pool radius (*R = R_h_ − t*) leads to a maximum whose position depends on the mismatch. In formula [29]:

(6) $$\frac{\chi }{\phi }=kT\frac{\varepsilon \varepsilon_{0}}{2\pi \eta }\frac{R_{h}-t}{R_{h}^{4}}.$$

where *ϕ = ϕ_w_* + *ϕ_s_*, *ε* is the relative dielectric constant and *ε*_0_ the vacuum permittivity.

#### 2.2.3. Dynamic Light-Scattering

Dynamic Light-Scattering (DLS) is one of the most applied techniques to study properties of liquid colloid systems. It is also known under the acronyms PCS (Photon Correlation Spectroscopy) or, in the past, QELS (Quasielastic Light-Scattering). It is, basically, an optical diffusion measuring technique. Commercially available instrumentations are relatively low-cost and allow fast and user-friendly determination of diffusion coefficients in a wide range of colloidal samples (often the output is directly the size obtained by inversion of Equation (2)). In general, DLS is not the technique of choice for the study of all µEs; however, it can be very effective in the study of simple cases of systems made of discrete aggregates (o/w or w/o) dispersed in a well-defined continuous bulk.

DLS measures time-dependent fluctuations in the light scattered intensity that are analyzed by a correlator. The output is the intensity autocorrelation function (ACF) whose rate of decay reflects the fluctuation modes of concentration in the system [30]. Since the ACF is dominated by fluctuations of the concentration of particles undergoing Brownian motion in a continuous medium, its rate of decay is associated directly with a diffusion coefficient *D_m_* that depends on the motion of both particles and solvent molecules. Strictly, *D_m_* is the mutual (or collective) diffusion coefficient and it is different from the self-diffusion coefficients measured in dNMR [30]. Note that for a simple o/w µE, one measures three self-diffusion coefficients (*D_w_*, *D_oil_*, *Ds*) and one single *D_m_*. For dispersed particles such as o/w or w/o µEs, at infinite dilution, the *D_m_* measured by DLS and the self-diffusion coefficient of the particles coincide and are equal to the diffusion coefficient entering the Stokes–Einstein relation shown in Equation (4). Additionally, in the case of a mutual diffusion coefficient, it is possible to write the concentration dependence as a Taylor series [31]:

(7) $$D_{m}=D{}^{\circ}\left(1+k_{DLS}\phi \right),$$

where the constant *k_DLS_* depends on the interparticle interactions. In a w/o µE, the continuous phase is an apolar oil and the electrostatic interactions are negligible so that the droplets are reasonably approximated as hard-spheres. An analogous situation is found for o/w µEs made by nonionic surfactants. In the case of hard-sphere interactions, the constants for self and mutual diffusion are known: for self-diffusion, *k_NMR_* = −2.1, and for mutual diffusion, *k_DLS_* = +1.45. Using such values, it is possible to evaluate the reverse micelle size by combining Equations (4) and (5) for dNMR, or Equations (4)–(7) for DLS. Possibly, having measurements with both techniques allows checking the validity of the hard-sphere assumption [22,32]. The situation becomes much more complicated in the case of o/w µEs made of charged surfactants because, in such cases, long-range repulsive interactions are expected. Accurate sizing of the o/w micelles and quantification of the strength of the interactions can be obtained by comparing the self (from dNMR) and mutual diffusion coefficients (by DLS) as detailed in references [33,34].

A bonus of DLS is that, during the experiments, the average intensity of scattered light is also measured. In the case of micelles smaller than the wavelength of the light used (a condition met by definition of µEs), this quantity is directly related to the osmotic compressibility and, therefore, to interparticle interactions (excluding the hydrodynamic interactions that plague the analysis of diffusion data) [31,32].

#### 2.2.4. Small-Angle Scattering

The analysis of the scattering profile collected at small angles for X-rays (SAXS) and neutrons (SANS) has been widely used in µEs to probe their microstructure in terms of morphology (spherical, cylindrical, flat, etc.), length scale(s) of the aggregates, and of changes obtained when formulation conditions change [35,36]. In these experiments, the intensity is represented as a function of the scattering vector (also called momentum transfer) $q=\frac{4\pi }{\lambda }sin\left(\frac{\theta }{2}\right)$, where *λ* is the wavelength of radiation and the scattering angle *θ* is the real experimental variable. The scattering vector is an inverse length, and 2*π*/*q* is the typical real space length probed by the radiation [37]. SAXS can be performed with an inhouse set-up, although the availability of synchrotron light sources has made it possible to perform experiments with incident brightness and intensity many orders of magnitude higher than that of X-rays produced in conventional laboratory instrumentations. On the other hand, the access to large-scale facilities is mandatory to perform SANS experiments, which, when coupled with a suitable choice of isotopic contrast matching between the oil and the water phase, is a unique way to probe the details of surfactant interface.

In the cases in which the µEs are described by well-defined particles in a continuous homogeneous phase (e.g., o/w and w/o µEs) it is customary to describe the scattering profile factorizing it as the product of two angular functions considering the two interferences in scattered radiation: intraparticle interference (*P*(*q*), the form function whose analytical form depends on the particle shape) and the interparticle interferences (*S*(*q*), the structure-function) according to:

(8) $$I\left(q\right)=N_{p}\Delta \rho^{2}v_{p}^{2}P\left(q\right)S\left(q\right)$$

where *N_p_* is the number density of particles of volume *v_p_* and Δ*ρ* is the contrast. For discrete particles, one can retrieve geometrical parameters (es length, cross-sectional radius) by fitting the experimental data to Equation (8) using suitable analytical models for *P*(*q*) and *S*(*q*). Another approach is to find the best geometry accounting for the “pair distance distribution function” obtained by the model-free Indirect Fourier Transform of the scattering data [38].

The situation becomes much more complicated in the realm of the bicontinuous µEs, where any discrimination between intraparticle and interparticle interferences is useless. Important insight on the structure of “balanced” µEs has been obtained by comparison of the scattering profile with sophisticated simulations of the three-dimensional arrangement of oil/water domains (e.g., Voronoi tessellation, MD simulation, etc.). Details on such approaches can be found in a recent review [39]. Here, we just mention a couple of model-free approaches that can give access to useful pieces of information also in the case of bicontinuous µEs. Often in these systems, the scattering curve presents a maximum due to the existence of an average distance between nanoscopic domains (differing in scattering length density). The scattering vector *q_peak_* at which the maximum is located denotes a characteristic distance:

(9) $$d^{*}=\frac{2\pi }{q_{peak}}.$$

Very often, such a characteristic distance can be identified with the persistence length *ξ* entering Equations (2) and (3). Moving in the region of high scattering vector (the so-called Porod’s regime), independently from the particle shape (if any), the asymptotic behavior of scattering intensity depends only on the specific area ∑ according to

(10) $$\underset{q\to \infty }{lim}I\left(q\right)=\phi \Delta \rho^{2}\sum q^{-4}.$$

Accordingly, in the so-called “Porod’s plot” of *q*^4^*I*(*q*) vs. *q*, one expects an asymptote at high enough *q* from which, knowing the contrast and the volume fraction, the interfacial area density ∑ can be determined. Strictly, the Porod’s limit holds for two-phase systems characterized only by two scattering length densities. A µE is, at least, a three-domains system (oil, water, and interface) and to observe the Porod’s limit is necessary that the surfactant and one of the other phases (oil or water) share the same scattering length densities (a situation that can be achieved in SANS by a suitable isotopic matching).

#### 2.2.5. Electron Microscopy (EM)

Microscopy is the only technique that allows us to directly observe colloidal systems in their size and shape. While scattering and NMR techniques use different probes and models to characterize colloids, micrographs are more tangible evidence of the structure of complex fluids [40].

For the direct visualization of objects with sizes below 100 nm, such as the case of microemulsions, electron sources are needed. Both scanning and transmission electron microscopies (SEM and TEM) involve extreme conditions. The sample, before entering the high vacuum chamber, must turn into solid-state in order to not be damaged or sucked by the pump. According to the different treatments the sample may undergo, it is possible to define several EM techniques: the freeze-fracture–transmission EM (FF-TEM), the cryo–field emission scanning EM (cryo-FESEM), and the cryo–transmission EM (cryo-TEM) [40].

Because of the invasive sample treatment, the interpretation of micrographs can be tricky, and the choice of the best technique is relevant. The freeze-fracture preparation minimizes the risks of artifacts and represents the best choice for the case of w/o and bicontinuous microemulsions, which are very difficult to be prepared by cryo-TEM [41,42,43]. On the other hand, cryo techniques better achieve sample protection [43,44,45]. The combination of both techniques enhances the reliability of the information and minimizes the artifacts, moreover for complete quantitative characterization of the system one needs the results from the other techniques previously discussed [41,46].

#### 2.2.6. Composition Perturbation

**Figure 4:**
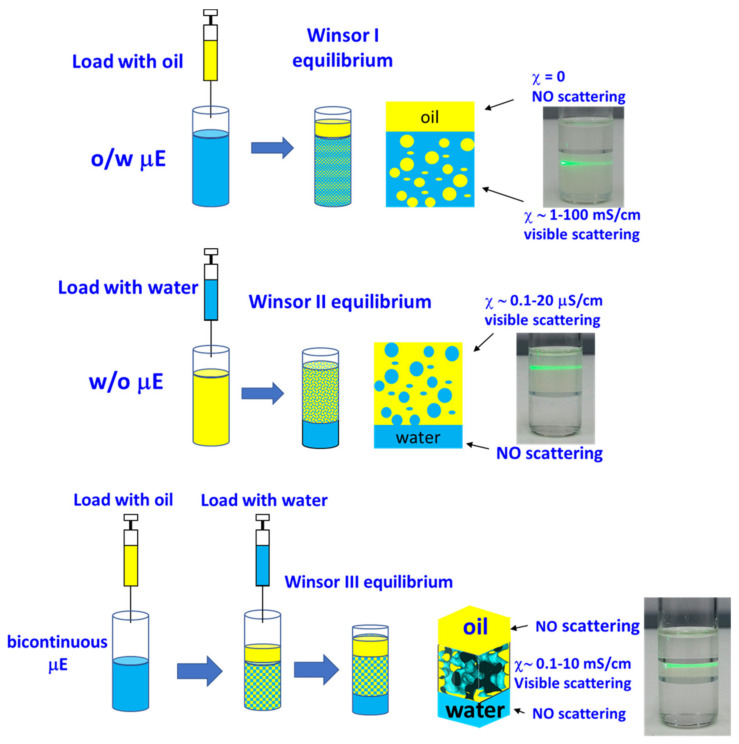
This scheme shows the fates of different perturbed μEs. Adding oil to oil-in-water (o/w) μE leads to a Winsor I regime, while when loading with water w/o μE, a Winsor II regime occurs. Bicontinuous μEs will reject both oil and water when loaded forming a Winsor III system. The photos on the right side display the behavior of different systems hit by a laser pointer. *χ* denotes the conductivity.

Besides the use of instrumental techniques, there is another approach, based on the response of the system to (small) perturbation in the composition that can give insight on the microstructure of the µEs (see Figure 4). The thermodynamic justification of the observed behavior is postponed to Section 5.

Loading a µE with oil or with water should have a different impact depending on what is its microstructure. For example, loading with water an o/w µE should, merely, dilute the swollen micelles without affecting the system’s stability. On the other hand, if the very same system is loaded with oil, we expect an increase in micelle size and therefore in the total interfacial area and a change in the interface curvature. This has an energy cost, and, at a certain point, the system simply refuses to solubilize further oil that remains as an excess phase. This “emulsification failure”, therefore, leads to a phase separation between o/w µE and excess oil that is called the Winsor I phase equilibrium. A specular situation is observed in the case of w/o µE for which the emulsification failure of water leads to a phase separation between w/o µE and excess oil that is called the Winsor II phase equilibrium.

Bicontinuous µE can be swollen by both oil and water but, also in this case, there are limits to the aomunt of liquids they can incorporate without turning into disconnected µEs. Additional oil and water are rejected into excess coexisting phases leading to the Winsor III three-phase equilibrium.

The location of water and oil excess phases can be usually associated with the difference in density. The presence of the µE gives rise to high light-scattering so that the beam of a laser pointer is visible, while in the case of pure oil or water, it is not. In addition, one can implement such observation with the conductivity measurements described in Section 2.2.2.

**Figure 5:**
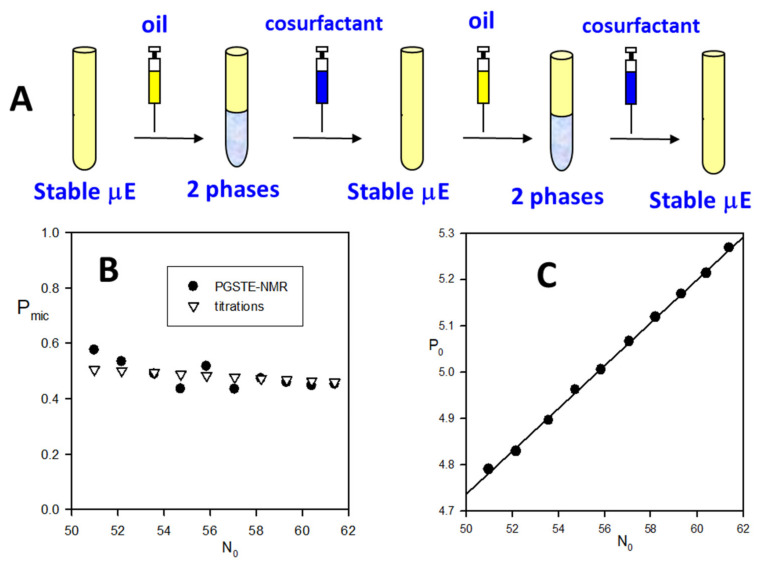
(**A**) Steps of Schulman’s titration: subsequent additions of oil and cosurfactant, respectively, destabilize and stabilize the w/o μE. (**B**) The *P_mic_* (fraction of cosurfactant in micelle) vs. *N*_0_ (oil/surfactant mole ratio) plot shows how the cosurfactant concentration remains constant during the titration; closed dots are from dNMR experiments while open triangles are from the titration of (**C**). (**C**) Representative Schulman’s titration: *P_o_* (alcohol/surfactant molar ratio) vs. *N*_0_ plot. The straight line is the best fit to Equation (11). The intercept is the minimum cosurfactant/surfactant ratio for the uE and the slope represents the bulk composition; the open triangles of (**B**) are *P_mic_* = intercept/*P_o_*. Adapted with permission from [49]. Copyright (2004) American Chemical Society.

Care must be taken that the perturbation in the composition should not alter catastrophically the partition equilibria. For example, at the dawn of the µE science, Bowcott and Schulman were able to form w/o µEs from a water-in-oil (macro)emulsion by titration with medium-chain alcohol [47]. A fraction of the alcohol adsorbs on the interface, decreasing the *γ* (and optimizing the surfactant packing) so that the (macro)emulsion turns into a microemulsion. In this respect, the cosurfactant role of the alcohol is like that of the surfactant, the main difference being that it has a low affinity for the interface. The amount of cosurfactant at the interface is ruled by the partition equilibrium with bulk oil. Indeed, by loading with oil a limpid w/o µE, it drains cosurfactant from the interface, and finally destabilizes the system that expels water. The single-phase µE can be restabilized by the addition of a requisite amount of alcohol. This is the basis of a simple method to determine the minimum amount of cosurfactant at the interface required to form a w/o µE. The procedure of the so-called Schulman’s titration is schematized in Figure 5A. A limpid w/o µE stabilized by cosurfactant is destabilized (becomes turbid) by dilution with a known amount of oil; then, the minimum amount of cosurfactant required to restore a transparent µE is recorded. The procedure is repeated several times. All the limpid samples through the titration procedure have a constant cosurfactant concentration in the bulk and interface [48]. This was demonstrated by the dNMR results of Figure 5B [49], where the fraction of cosurfactant in the reverse micelles is shown to remain constant upon changing the molar ratio oil/surfactant = N_0_ along with the titration. The mass balance requires that:

(11) $$\frac{n_{c}}{n_{s}}=P_{0}=P_{0}P_{mic}+{\left(\frac{n_{c}}{n_{o}}\right)}_{bulk}\frac{n_{o}}{n_{s}}.$$

where *P*_0_ = *n_c_*/*n_S_* is the mole ratio cosurfactant/surfactant, *P_mic_* is the fraction of cosurfactant in the reverse micelle and ${\left(\frac{n_{c}}{n_{o}}\right)}_{bulk}$ is the molar ratio cosurfactant/oil in the continuous phase. The data collected along the titration are used to construct graphs by plotting the molar ratio alcohol/surfactant (*P*_0_) vs. the molar ratio oil/surfactant as shown in Figure 5C.

According to the above equation, the data of Schulman’s titration can be easily fitted to a straight line whose intercept is the minimum cosurfactant/surfactant ratio at the interface compatible with a single-phase μE while the slope gives the corresponding continuous bulk composition.

**Figure 6:**
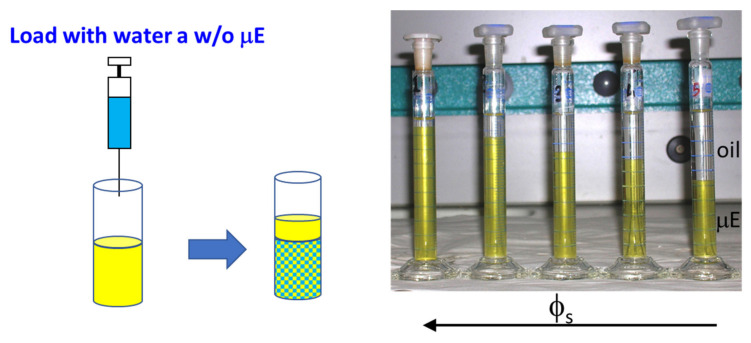
In the example on the left, an asymmetric bicontinuous μE is loaded with tiny amounts of water. Above a certain water content, the system phase separates into a dense (and viscous) μE in equilibrium with oil. On the right, in the case of the system water/soy-bean lecithin/pentane, the phase separation takes place for molar ratio water/lecithin = 7; the test tubes represent a series of samples at a constant molar ratio of water/lecithin = 7 and different *ϕ_s_* ranging from 0.03 (**right**) to 0.08 (**left**). For *ϕ_s_* > 0.115, the system does not phase separates.

We conclude such an overview introducing a further phenomenology that can be observed loading a stable μE with its dispersed phase. In Figure 6, we present the case of a w/o μE that is loaded with water and, when the water/surfactant ratio exceeds a critical value, the systems expel oil leading to a peculiar phase separation between a dense μE and a very dilute one (almost pure oil). Keeping the water/surfactant ratio constant, the response of the system to a change in surfactant concentration is interesting: at low surfactant concentrations, the system separates, expelling a large amount of solvent (oil). A further increase in *ϕ_s_* increases the surfactant solubility until a point where only one stable phase is observed [50,51] (sometimes such behavior is called re-entrant phase separation).

The thermodynamic roots of this behavior will be discussed in Section 4 further on, here, we anticipate that in the presence of this kind of phase separation, it is very likely that the system forms asymmetric bicontinuous networks

## 3. Microemulsion Phase Behavior

According to what we just said, the phase behavior of µE systems strongly correlates with their microstructure. The determination of phase diagrams (mapping the composition at which different structures exist) within these water(salt)–oil–surfactant–alcohol systems is, therefore, very important. Usually, the discussion of the phase diagrams is introduced after the presentation of a theoretical model. Since one of the scopes of the present review is the comparison of different theoretical models (in Section 5), here we limit ourselves to a schematic illustration of the representation mode of phase diagrams depending on the variables involved and the typical phenomenology observed.

By far, the most comprehensively studied class of truly ternary μEs are those based on a nonionic surfactant of the alkyl oligoethylene oxide type with the general structure:

CH_3_–(CH_2_)*_N_*_−1_(OCH_2_CH_2_)*_J_*OH  (C*_N_*E*_J_*)

**Figure 7:**
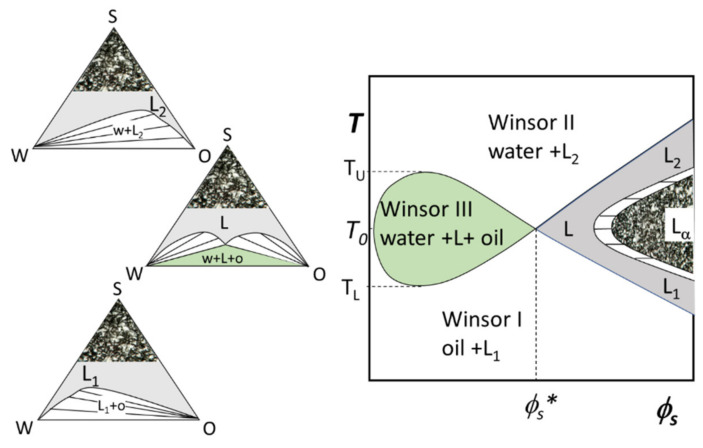
On the right, schematic bidimensional Kahlweit’s “fish-plot” phase-diagram at *ϕ_w_* = *ϕ_o_* for ethoxylate-based ternary microemulsion around the balance temperature *T*_0_. One-phase μE (in gray) is found only above ϕs* in a region with the shape of a fish-tail. The fish-shaped body (in green) contains three-phase systems (Winsor III). L_α_ denotes a lamellar liquid crystalline phase. On the left side, three corresponding isothermal phase diagrams are reported. From bottom to top: *T* < *T*_0_, *T* = *T*_0_, *T* > *T*_0_.

Typical values are *N* = 10–16 and *J* = 3–8. The phase properties and phase behavior of these surfactants in ternary microemulsions are strongly temperature-dependent and have been studied extensively [52,53]. To explore the strong T-dependence with the minimum number of samples, and because of the paramount importance that balanced μEs have, one often fixes equal amounts of oil and water (*ϕ_o_* = *ϕ_w_*) and changes the amount of surfactant and temperature. The corresponding phase diagram is called the Kahlweit’s “fish-plot” and is schematically illustrated in Figure 7. In such a *T* vs. *ϕ_s_* phase diagram, one can determine the minimum required surfactant concentration to solubilize equal amounts of water and oil, ϕ_s_*. The lower ϕ_s_* the more efficient is the surfactant. Just above ϕ_s_* and around the so-called balance temperature *T*_0_ (corresponding to the phase inversion temperature, PIT, for macroemulsions stabilized by the same surfactants), one can find a single-phase μE system (solubilizing all the volumes of oil and water).

The single-phase μE region, resembling a fish-tail, is shaded in gray in Figure 7. By definition, all the μEs are isotropic liquid phases, for this reason, they are indicated in the phase diagrams by the letter L (for liquid) with the subscript that considers the connectivity of the components: L_1_ stands for o/w, L_2_ stands for w/o. In the neighborhood of the point at *T*_0_ and ϕ_s_*, the μE is bicontinuous (denoted simply by L). Increasing *T* favors reverse curvatures so that the microstructure evolves continuously toward reverse micellar aggregates (L_2_). The opposite situation takes place lowering *T* below *T*_0_. At high surfactant concentrations (*ϕ_s_* ϕ_s_*), the reverse micelles (L_2_ at high-*T*) and the direct micelles (L_1_ low-*T*) domains are separated by a lamellar liquid crystalline phase (denoted as L_α_). Decreasing the surfactant concentration below ϕ_s_*, the system becomes multiphasic. The multiphase region at *ϕ_s_* ϕ_s_*, for equal amounts of oil and water, is of paramount importance in the µE study because it is where the simple visual inspection of samples allows understanding the effect of an intensive parameter (e.g., the temperature in the case of C*_N_*E*_J_* surfactants) on the surfactant spontaneous curvature. If the surfactant dilution takes place at the balance temperature, not all water and oil can be solubilized and there is a Winsor III separation with oil and water coexisting with a bicontinuous μE. This three-phase region (the fish-body) is circumscribed to the range *T_L_* ≤ *T* ≤ *T_U_*. For temperatures higher than *T_U_* the interfacial curvature is highly curved toward the water and discrete reverse micelles (L_2_) in equilibrium with excess water are found (Winsor II equilibrium). Usually, μEs stay on top of the water, so that in some papers such a two-phase situation is denoted as $\bar{2}$.

Speculatively, for *T* *T_L_* the interfacial curvature is highly bent toward oil and a Winsor I two-phase equilibrium takes place (sometimes denoted as $\underline{2}$).

Relaxing the constraint of fixed *ϕ_o_ = ϕ_w_*, the representation of phase behavior of three components requires isothermal ternary phase diagrams like the ones shown in Figure 7 (the reader can find interactive apps to familiarize with this sort of representation on the open-access book by Abbott [54]). Every point within the single-phase region (gray area) corresponds to a different µE composition. At *T* *T_U_* and at *T* *T_L_*, the μEs can also coexist with excess dispersed phase (white lenses in the figures). Any system whose overall composition lies within these two-phase regions will exist as two phases whose compositions are represented by the ends of the straight “tie-lines”. Every point on a particular tie-line has identical coexisting phases but different relative volumes. The phase behavior below *T_L_* is characterized by large one-phase regions formed by o/w μE (L_1_) that in the two-phase region is in equilibrium with almost pure oil. Indeed, all the tie-lines have one extreme in the corner oil. The opposite holds at *T* above the *T_U_* where tie-lines denote a Winsor II equilibrium between almost pure water and w/o μE (L_2_). The phase diagram at the balance temperature (*T*_0_) contains a region corresponding to the Winsor III equilibrium (green). According to the phase rule, at constant temperature and pressure, this region is invariant in composition. This region has, therefore, a triangular shape and any system within is formed by three phases whose compositions are given by the triangle apexes. Moving within such a region does not change the phases composition but only their relative amounts (again, the interested reader is referred to the app [55] from reference [54]).

**Figure 8:**
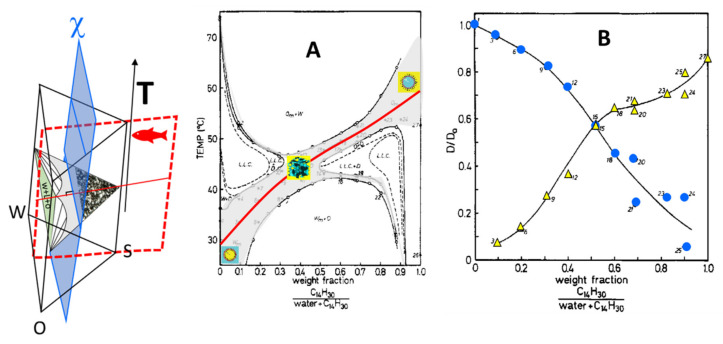
(**Left**) Example illustrating the ternary phase prism. The “fish cut” at *ϕ_o_* = *ϕ_w_* is shown as a red dashed plane; the “*χ* cut”, defined by a constant *ϕ_s_* is represented in blue. (**A**) Experimental “*χ* cut” phase diagram of the C_12_E_5_-water-tetradecane system at *ϕ_s_* = 17%. The isotropic liquid phase (µE) is shaded in gray and LLC denotes the lamellar liquid crystalline phase. The red curve denotes the composition-*T* path from o/w to w/o μE. Adapted with permission from [56]. Copyright (1986) American Chemical Society. (**B**) Relative self-diffusion coefficient of water (blue dots) and tetradecane (yellow triangles). The samples have been collected along the red path in (**A**). Adapted with permission from [56]. Copyright (1986) American Chemical Society.

To describe the complete behavior, including the T-effect, even of the simplest water–surfactant–oil systems, one should have ternary phase diagrams at each temperature stacked in the form of a prism-like the one shown in Figure 8 that accounts for four-variables (three compositions and the *T*). In this respect, the two-variable fish-plot of Figure 7 can be considered the intersection (in jargon the “cut”) of this prism with a vertical plane passing from the surfactant apex and the points at *ϕ_o_* = *ϕ_w_* (the red dashed plane in the example in Figure 8).

Another informative cut is the section at constant surfactant concentration through the phase prism often called χ-cut introduced by Shinoda (the blue plane in the example of Figure 8). In this set of experiments, the surfactant concentration is kept constant while the water/oil ratio and the temperature are changed. When the surfactant concentration is chosen, as in the example in Figure 8, above ϕs* but below the onset of the lamellar phase, the single-phase µE occupies a region like the one shown in gray in Figure 8A. This is the experimental “*χ* cut” phase diagram of the C_12_E_5_-water-tetradecane system [56] where one observes a µE channel extending from the waterside at *T* *T*_0_ (*T*_0_ = 48 °C) to the oil side at *T* *T*_0_. There is also a second, narrower channel having the opposite orientation that crosses the main µE channel around *T* = *T*_0_ such that the overall μE region has a *χ*-shape. A peculiar feature of this phase diagram is its C_2_ symmetry. Rotating 180 ° around the point *ϕ_o_* = *ϕ_w_*, *T* = *T*_0_, the same phase diagram returns. The microstructure evolution moving along the main channel (the red path in Figure 8A) can be unambiguously understood by observing the self-diffusion coefficients (diffusivities) of water and oil according to the discussion in Section 2.2.1. To remove the trivial T-dependence in the diffusion of water and tetradecane, Figure 8B shows the relative diffusion values, i.e., diffusivity measured in the μE, D, divided by the diffusivity of the pure liquid at the same temperature *D*_0_. At high water content $\left(\frac{\phi_{o}}{\phi_{o}+\phi_{w}}0.2\right)$ and low temperature, the water diffusivity is close to the one of neat water while the diffusivity of oil is strongly reduced. This is the evidence that oil is confined in closed domains of o/w μE. At the opposite side of the path (high *T* and large $\frac{\phi_{o}}{\phi_{o}+\phi_{w}}$), the oil diffusion is almost “free” while water, being secluded within the reverse micelles water pool, has a very low diffusivity. At *ϕ_w_*~*ϕ_o_* and at temperatures close to *T*_0_, the relative diffusion of both solvents is around *D*/*D*_0_~2/3. This is the theoretical value for reduced diffusion in a random array of the planar channel and, therefore, a hint that the interface in these balanced μE is effectively flat.

The very narrow channel going from high *T* at low *ϕ_o_* to low *T* at high *ϕ_o_*, corresponds to the so-called “sponge phase” (usually labeled L_3_) [23]. In contrast to the bicontinuous μEs that are formed by surfactant monolayers separating oil and water domains, the “sponge phase” is formed by bilayers separating two domains of the same solvent. These exotic μEs could be imagined as the tubular (and interconnected) version of the vesicles sketched in Figure 1C. At higher temperatures and higher water content, a normal, oil-swollen bilayer separates two water domains (labyrinths) that do not have any contact point. Therefore, L_3_ is strictly a tricontinuous phase with a continuous surfactant–oil domain and two distinct water labyrinths. At the opposite side of the channel (low *T* and high *ϕ_o_*) reverse water-swollen bilayers separate two oil labyrinths.

The situation in the case of real-world μEs is much more complicated because almost invariably they require the addition of further components (other surfactants, cosurfactants, linkers [57]). This poses enormous problems even in the simple representation of the phase behavior.

In order to formulate μEs with ionic surfactants very often one must load the system with cosurfactants (e.g., pentanol, hexanol) and tune the ionic strength of the aqueous phase. The double chain anionic surfactant AOT (sodium bis(2-ethylhexyl) sulfosuccinate) is one of the few ionic surfactants that form μEs without cosurfactants in a wide range of composition and temperatures. Using AOT it is possible to treat the system as a pseudoternary system brine–surfactant–oil and to study the interplay between temperature and ionic strength as determinants of the microstructure. Such a complex phenomenology has been quantitatively accounted for by the model proposed by Wennerström and coworkers in [57] (where one can find also the references to previous experimental studies).

For μEs based on ionic surfactants, the curvature of the interfacial film depends strongly on the repulsion among the surfactant charged headgroups. Such an effect tends to favor curvatures toward the oil (how much such a propensity is effective depends on the interactions among the tails). Another important effect is that such repulsive interaction increases the rigidity of the surfactant film favoring the formation of lamellar phases instead of bicontinuous μEs. In this respect, it is not surprising that increasing the ionic strength (by adding salt or by increasing the ionic surfactant) one screens the electrostatic interactions, making the interface more flexible and less curved toward the oil film. What is less intuitive is that the higher the temperature, the higher the magnitude of the electrostatic interaction [57]. The combination of these two effects explains the following main features of ionic surfactants phase behavior:

- o/w μEs are found at high temperature and w/o μEs are found at low temperatures; this is the contrary of the trend found for nonionic surfactants.
- The lamellar phase is found at *T* *T*_0_ because the interface curvature and stiffness decrease with increasing the ionic surfactant concentration but increase with the temperature.
- For the same surfactant, the balance temperature *T*_0_ increases with the added salt concentration for the same reason than above.
- As a consequence, the “fish” in the Kahlweit’s plot is “tilted” with the tail higher than the body.
- The symmetry observed for nonionic surfactants in the χ-plot is lost in the case of ionic surfactants. Exchanging oil and water changes the ion concentration (besides the balanced condition *ϕ_o_* = *ϕ_w_*).

## 4. Cylindrical Aggregates: Living Polymers vs. Living Networks

**Figure 9:**
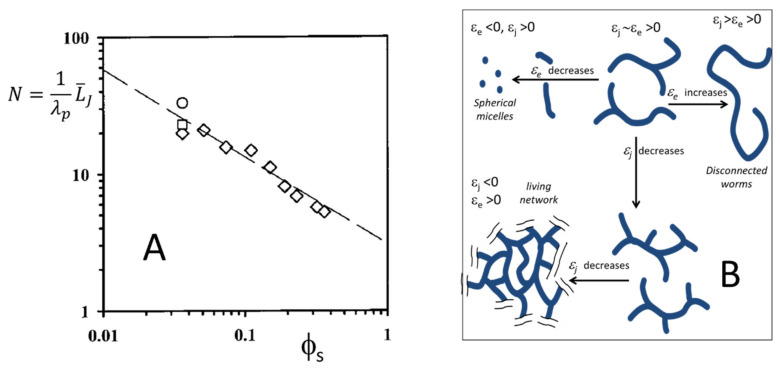
(**A**) μE water/soy-bean lecithin/isooctane at constant mole ratio water/lecithin = 2: plot of the number of Kuhn lengths, *N*, connecting two junctions as a function of *ϕs*. The line is the best fit to the power law ${\bar{L}}_{J}\propto \phi_{s}^{-\alpha }$; α = 0.55 ± 0.07 in agreement with Equation (14). Adapted with permission from [18]. Copyright (2001) American Chemical Society. (**B**) Scheme of the morphological changes occurring upon tuning the endcaps (*ε_e_*) and junctions (*ε_j_*) free energy with respect to the reference cylindrical shape with null energy. Adapted from [51] with permission from the PCCP Owner Societies.

We mentioned before (Section 2.1) that among the shapes the surfactant film can attain to seclude the dispersed phase, there is one of a cylindrical type. Spontaneous self-assembly of surfactants forming a cylindrical micelle implies that such a shape minimizes the free energy of the system. In this respect, the endcaps of the cylinder must represent a sort of defects associated with a high free energy penalty. This means that the fusion of *n* rods (2 × *n* endcaps) to form a single cylindrical object (only two endcaps) is energetically favored. Accordingly, simple (mean-field) theoretical models predict that these cylindrical aggregates growth in length forming very long and flexible micelles with an average contour-length ($\bar{L}$) proportional to the square-root of surfactant concentration (see part three of [58]):

(12) $$\bar{L}∼\sqrt{\phi_{s}}\times e^{\varepsilon_{e}}$$

where the proportionality constant depends exponentially on *ε_e_* (the endcap energy relative to the central moiety in units of thermal energy kT). Being very long and flexible these micelles share many features with the polymer solutions and are therefore referred to as polymerlike or wormlike micelles. To have a cylindrical micelle it is not necessary to have a microemulsion. Actually, most of these systems are simple single tail surfactants aqueous solutions at high ionic strength. However, the concepts required to understand the roots of the parameter *ε_e_* in Equation (12) are intimately linked to the theories developed for μEs and there are several interesting examples of μEs made by wormlike aggregates [16,17,51]. As in the case of polymers, the wormlike micelles become entangled, above a critical concentration, and form a transient network. However, for polymers, the size distribution is quenched while, according to Equation (12), wormlike micelles change their length with concentration and with all the parameters that can have an effect on the endcap penalty *ε_e_* (*T*, ionic strength, composition and so on). The *ϕ*-dependence of $\bar{L}$ has been exploited to get the scaling laws for zero-shear viscosity, plateau modulus, and the overlap concentration of wormlike solutions by improving the reptation theory for polymer solutions, [59]. In addition, micelles are dynamic entities dissociating and recombining with time, and to manage their response to the mechanical stress, the characteristic time of breaking/recombination (*τ_break_*) must be considered. In this context, these systems are also referred to as “living polymers”. In the fast breaking limit, the stress relaxation is single-exponential with a characteristic time *τ* given by [60]:

(13) $$\tau ∼\sqrt{\tau_{break}\tau_{rept}}$$

where *τ_rept_* is the reptation time. Accordingly, single relaxation time in rheological measurements is often experimentally observed despite the high polydispersity of the micelle size. In some cases, the cylindrical micelles can form branches or junctions and a further length scale becomes significant: the average distance between junctions ${\bar{L}}_{J}$. When the density of such junctions becomes high (${\bar{L}}_{J}$ becomes short), the systems can be considered the micellar counterpart of cross-linked polymer networks but with reversible connections. The characterization and the modeling of such “living networks” is yet a challenge but different theoretical approaches agree in foretelling that the distance between branches scales as [50,61,62,63]:

(14) $${\bar{L}}_{J}∼\frac{e^{\varepsilon_{J}}}{\sqrt{\phi_{s}}}.$$

where *ε_J_* is the junction energy (with respect to the cylindrical case). Accordingly, increasing the *ϕ_s_* reduces the ${\bar{L}}_{J}$ as shown in Figure 9A where the experimental dependence of the ${\bar{L}}_{J}$ on surfactant, the volume fraction is reported for a μE made of trace amounts of water, lecithin, and isooctane [18]. The experimental data ($N={\bar{L}}_{J}/\lambda_{p}$, where *λ_p_* is the micelle persistence length) have been achieved by dNMR using an approach tailored to study wormlike micelles (for an introduction on these approaches, see [64]). The increase in the branch density could be strong enough to drive a phase separation between a fully branched living network and a dilute micellar phase (reminiscent of a cross-linked gel in equilibrium with excess solvent) [51].

The theories describing the living network predict a couple of counterintuitive characteristics [50,61,62,63]:

- The phase separation is re-entrant, i.e., the μE phase separates at low *ϕs* but is stable at high surfactant concentration (as in the case of samples in Figure 6);
- Micellar junctions lead to a reduction in viscosity because the branches can slide without restrictions along the micellar contour (for experimental confirmation, see [65]).

The overall scenario embracing disconnected small micelles, long wormlike micelles and living networks can be discussed in terms of free energy penalty associated with different local shapes: cylindrical (taken as the reference and thus with null energy cost), (emi)spherical endcaps with energy *ε_e_* and the saddle-shaped junction with energy *ε_J_*. Figure 9B is a schematic representation of the morphological evolution of these systems. High values of both *ε_J_* and *ε_e_* values favor disconnected wormlike micelles. Any change in the intensive variable that reduces *ε_e_* promotes a reduction in the length of this micelle until for *ε_e_* 0 only spherical micelles are allowed. On the other hand, for *ε_J_* 0 the formation of branches is favored and for *ε_J_* 0 fully branched living networks form spontaneously.

The overall scenario embracing disconnected small micelles, long wormlike micelles and living networks can be discussed in terms of free energy penalty associated with different local shapes: cylindrical (taken as the reference and thus with null energy cost), (emi)spherical endcaps with energy *ε_e_* and the saddle-shaped junction with energy *ε_J_*. Figure 9B is a schematic representation of the morphological evolution of these systems. High values of both *ε_J_* and *ε_e_* values favor disconnected wormlike micelles. Any change in the intensive variable that reduces *ε_e_* promotes a reduction in the length of this micelle until for *ε_e_* 0 only spherical micelles are allowed. On the other hand, for *ε_J_* 0 the formation of branches is favored and for *ε_J_* 0 fully branched living networks form spontaneously.

## 5. Modeling the Microstructure as Rationale for µE Formulation

### 5.1. Packing Parameter and HLB

Over the years, the development of models to describe µEs has been raised as an attempt to formulate and optimize their geometries and properties.

The oldest of the models here described, which is still in use today, is the hydrophilic–lipophilic balance (HLB). It was initially introduced by Griffin in 1949 [66] as a tool to classify nonionic ethoxylated (C*_N_*E*_J_*) surfactants according to their hydrophilicity using a number scale between 0 and 20. The general rule of thumb (known as Bancroft’s rule) states that hydrophilic surfactants tend to give o/w emulsions and hydrophobic surfactants w/o emulsions. Therefore, the HLB theory proposes that in order to formulate a w/o emulsion, one should use a surfactant with an HLB number between three and six, while o/w emulsions should be done with surfactants with an HLB number between 8 and 18. There is experimental evidence that if a given brine/surfactant (s)/oil (s) system at high *ϕs* form an o/w μE, it will form, above the emulsification failure, an o/w emulsion. Analogously, systems forming w/o μE when mixed with water give rise to stable w/o emulsions. Balanced systems demulsify very fast. In this respect, the HLB is also a predictive tool for μE as well. The problem with HLB is that its predictive power is, sadly, very low (in addition the extension of the HLB number to nonetoxylated surfactants is questionable).

Later on, in 1976, Israelachvili et al. introduced the idea of a Critical Packing Parameter (*CPP*) [9]. This theory rationalizes the type of assemblies of pure surfactants in water depending on the shape of the surfactant. The concept of *CPP* emerges naturally in the case of *n_s_* molecules of surfactant in water self-assembled to give of *n_mic_* micelles. In the case of spherical micelles, the overall apolar volume can be defined in terms of chemical composition as *V* = *n_s_v* (where *v* is the volume of the surfactant tail) or geometrically as $V=n_{mic}\frac{4}{3}\pi R^{3}$ (where R is the radius of the apolar micellar core). Analogously, the overall polar/apolar area can be expressed as $A=n_{mic}4\pi R^{2}=n_{s}\alpha$ where *α* is the surface area per surfactant at the polar/apolar interface (the head area). Making the volume to surface ratio one correlates the apolar core radius to surfactant tail volume and head area according to $R=\frac{3v}{\alpha }$. If the system is made only by surfactant and water (i.e., it is not a μE), the radius cannot exceed the critical length *l*, the surfactant tail length, i.e., *R* ≤ *l*. Accordingly, the critical condition for the formation of spheres is that the dimensionless value defined below,

(15) $$CPP=\frac{v}{\alpha l}$$

must be *CPP* ≤ 1/3. Analogous calculations for a cylindrical micelle (neglecting the endcaps) gives *CPP* ≤ 1/2 while for lamellae the *CPP* is close to 1 (*V ≈ αl*).

**Figure 10:**
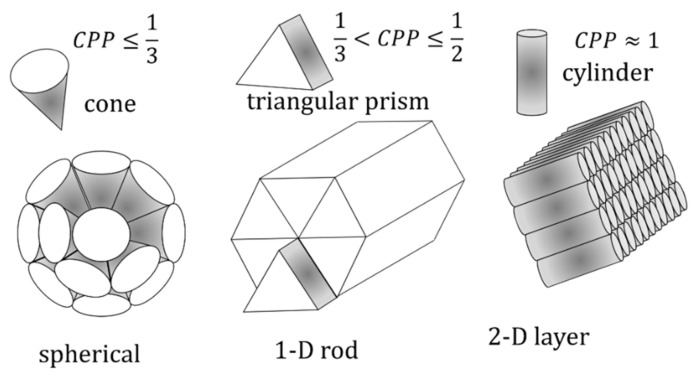
Correlation between the building block shape (quantified by the critical packing parameter) and the shape of the aggregates.

Basically, the *CPP* compares the actual tail volume with the volume of the hypothetical cylinder with a base *α* and a length *l*. As such it is an estimator of the molecular shape. The volume ratio between a given shape and the cylinder sharing the same base and height is 1/3 for a cone, 1/2 for a triangular prism and, by definition, one for the cylinder itself. The outcome of such reasoning is simple and powerful. Surfactants spontaneously self-assemble into the aggregates having shapes that allow for the best packing of the molecules as shown graphically in Figure 10. Cones can be efficiently packed, side by side, spherically but cannot be arranged to form an aggregate that grows in one dimension such as a rod (or a cylinder). To build a rod or a cylinder, one should use triangular prisms or cheese triangles, respectively. Finally, true cylinders are efficiently packed in the form of two-dimensional layers.

The effect of concentration can be grossly included in the model considering the packing constraint of the aggregates: For *CPP* ≤ 0.33, the surfactant forms classic micelles. When these spherical aggregates can no longer pack (the maximum volume fraction is 0.74) they are forced to form a hexagonal array of cylinders (0.5 *CPP* 0.33) that can pack up to a volume fraction of 0.91. Above such a limit only lamellar phases (*CPP*~1) can be found. Furthermore, the concept of *CPP* can be coupled with the dependence of head area α on the temperature, in the case of the nonionic surfactant and ionic strength in the case of ionic surfactants, to successfully rationalize the phase behavior of water/surfactant systems.

### 5.2. Measures of the Curvature of a Surface

**Figure 11:**
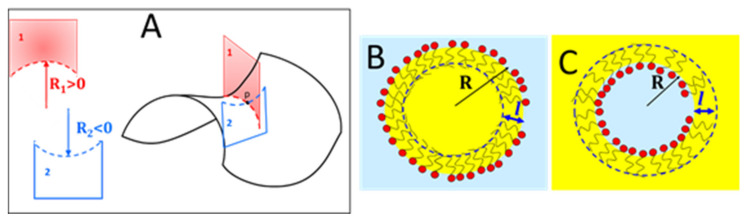
((**A**), right) Intersection of a surface with the orthogonal planes defining the principal curvatures. On the left are shown the corresponding plane curves (dashed) and the principal radii of curvature. (**B**,**C**) Views of the radius of curvature of the polar/apolar interface (*R*) and of the thickness (*l*) of the interfacial film for spherical o/w and w/o micelles, respectively.

Intuitively, spherical and cylindrical micelles and planar bilayers are characterized by different curvatures. Now it is appropriate to define and quantify the curvature of a generic surface. Consider a generic surface in the 3D space as the saddle depicted in Figure 11A. To define the curvature at the point *p*, take a plane normal to the surface passing through *p* (Plane 1 in the figure). Such a plane cut the surface defining a plane curve (dashed line in Figure 11A) characterized by its own curvature c_1_ defined as the reciprocal of the radius *R*_1_ of the osculating circle (best-fitting the curve); see the normal section on the left of Figure 11A. With respect to the point *p*, there are infinite normal planes corresponding to generally different curvatures. The principal curvatures (*c*_1_ = 1/*R*_1_ and *c*_2_ = 1/*R*_2_) are the maximum and minimum curvatures of this set of values. The normal planes corresponding to the principal curvatures are always perpendicular to each other like Planes 1 and 2 in Figure 11A. In the case of the saddle-shaped surface of Figure 11A, the radius of the principal curvatures stays on different sides with respect to the surface and have opposite signs. The side on which the radius, and thus the curvature, is taken as positive is chosen on the basis of an arbitrary convention. The principal curvatures can be combined to give two useful measures of the surface curvature:

(16a) $$The\;mean\;curvature\;H=\frac{c_{1}+c_{2}}{2}=\frac{1}{2}\left(\frac{1}{R_{1}}+\frac{1}{R_{2}}\right)$$

(16b) $$The\;Gaussian\;curvature\;K=c_{1}c_{2}=\frac{1}{R_{1}}\frac{1}{R_{2}}$$

Therefore, *H* has the dimension of a reciprocal length and *K* has the dimension of a reciprocal area.

Passing to simple shapes:

For a sphere $H=\frac{1}{R_{s}}$ and $K=\frac{1}{R_{s}^{2}}0$ (*R_s_* = sphere radius);
For a cylinder $H=\frac{1}{2R_{c}}$ and K=0 (*R_c_* = cross-sectional radius of the cylinder);
For a plane *H* = 0 and *K* = 0;
For a saddle shape as in Figure 11A, *R*_1_ and *R*_2_ have opposite sign, and in the case *R*_1_ = −*R*_2_, one as such that the mean curvature is null *H* = 0 and $K=\frac{1}{R_{1}R_{2}}$ 0.

### 5.3. Effective Packing Parameter

The *CPP* only considers systems formed by surfactants in water and cannot easily handle the presence of oil. This is because, in the presence of oil, the micelle is not constrained to have an apolar core having a size equal to the surfactant length (see the examples in Figure 1). Here, comes into play the effective packing parameter (*PP*), which considers, not only the simple geometrical considerations but also the interactions between head groups and those between tails. This model keeps the equation from the *CPP* equation (Equation (15)) but estimates, instead, an effective head group area *a* and an effective tail volume *V* that accounts for the apolar part of the molecule including the “penetrating oil”. For example, the interactions between head groups of ionic surfactants will be strongly affected by the presence of electrolytes, as *a* decreases with increasing electrolyte concentration. In the case of nonionic surfactants, the effective head group area decreases with increasing temperature, as the solvation sheath is diminished. On the other hand, the degree of oil penetration into the interface containing the hydrocarbon chains affects their effective volume.

For example, oil penetration increases as the length of the hydrocarbon chain of the oil decreases with respect to that of the surfactant [16,67]. Adding a cosurfactant such as dodecanol to a single-chain surfactant will also increase *V*, while the spacing between headgroups remains virtually unaltered since the polar OH group are very small [16].

Roughly, the effective packing parameter describes the shape of the interfacial film building block (surfactant + bound oil). As in the case *CPP*, one expects for *PP* 1 a wedge shape that favors direct structures; *PP* = 1 favors planar film and *PP* 1 favors reverse structures. However, such an interpretation is not sufficient to determine uniquely the interfacial geometry. In terms of *PP*, the molecular shape determines the local curvature of the interface and, considering the finite thickness of the interfacial film, the following relation between effective packing parameter and Gaussian and mean curvature can be demonstrated [11,68]:

(17) $$PP=\frac{V_{eff}}{\alpha l}=1+Hl+\frac{Kl^{2}}{3}.$$

where *H*, *K*, and the head area α are evaluated on the same interface. Note that the sign of *H* or, alternatively, the sign of *l* is dependent upon which side of the interface is taken as positive. The authors that introduced the packing parameter adopted, to apply Equation (17), the convention that *l* is always positive and the mean curvature is positive when is curved toward the polar region and negative otherwise (i.e., *H* 0 for reverse micelles and *H* 0 for direct micelles) [11]. This can cause some confusion because, in the μE field, the opposite convention is often adopted. A way to circumvent such a problem is to handle Equation (17) assuming *H* is always positive and considering whether *l* adds or subtract length to the radius of curvature, i.e., *l* 0 for direct micelles and *l* 0 for reverse micelles (see Figure 11B,C). Whatever the convention used, for direct structures *Hl* 0 and in the case of a spherical micelle, in the absence of oil constrained to have a radius equal to the surfactant length $\left(Hl=\frac{1}{R}l=\frac{1}{R}\left(-R\right)=-1\right),$ one retrieves the result that *PP* = 1/3 = *CPP*.

Equation (17) grasps the fact that, in the case of the microemulsion, the effective packing features of the surfactant do not fix the shape of the aggregates but only the combination of Gaussian and mean curvature interface. In other words, for a given effective packing parameter the self-assembled aggregate can change shape and size (i.e., *H* and *K*) as long as the combination 1 + *Hl* + *Kl*^2^/3 remains constant. For a given μE (at constant *T*) there is a surfactant conformation that has the easiest packing at the interface, the corresponding effective *PP* is the spontaneous packing parameter (*PP*°). If the term 1 + *Hl* + *Kl*^2^/3 is different from the *PP*°, the surfactant film is said to be “frustrated” and the film arranges itself, changing *H* and *K* in order to fulfill *PP*°.

The μEs formed by the cationic surfactant didodecyl-dimethylammonium bromide (DDAB) with different oils and water have been studied in detail [16,69,70]. At low water content, the system forms asymmetric bicontinuous w/o µE based on cylindrical water-filled conduits.

The cross-sectional radius of such reverse cylinder (*R_c_*) fulfills the requirement for a cylindrical geometry 0.5 ≈ *PP*° = 1 + *l*/*R_c_* (the last equality corresponds to Equation (17) for a cylindrical geometry). Water loading should increase the cylinder cross-sectional radius, *R_c_*, “frustrating” the film that instead changes into spherical reverse micelles of radius *R_s_* for which Equation (17) attains a value *PP* = 1 + *l*/*R_s_* + *l^2^*/*R_s_*^2^ closer to the *PP*°. Accordingly, there is a transition from cylindrical branched reverse micelles to spherical ones that explains the antipercolation transition in electric conductivity experimentally observed upon water addition. Anyhow, also for a spherical reverse micelle, the *PP* decreases with water loading until all the DDAB is involved in the formation of water droplets with optimal size. This water content corresponds to the emulsification failure. The incorporation of such a scenario of the *H* and *K* corresponding to the branch points and the dependencies on the hydration of *PP*° allows the quantitative prediction of the phase boundaries of the μE [69].

Lately, it has been proposed to translate such geometrical constraints into thermodynamic arguments. In analogy with the flexible surface model discussed in the following section, the free energy cost to bend the interfacial film (per unit area), hereafter bending energy density = *g*, was postulated to depend quadratically on the film “frustration” according to [71,72]:

(18) $$g=\frac{1}{2}k^{*}{\left(PP-PP{}^{\circ}\right)}^{2}.$$

where k* is a generalized bending constant. However, such an approach has been mainly limited to “rigid microemulsions” (k* 10*k_b_T*).

### 5.4. Flexible Surface Model

In the case of μEs made up of flexible surfactant films, one focuses on the interfacial film assumed to behave as a geometric surface with negligible thickness. The energy cost per unit area associated with elastic undulations to the lowest order is assumed to be a quadratic function of the principal curvatures:

(19) $$g=2\kappa {\left(H-H_{0}\right)}^{2}+\bar{\kappa }K=2\kappa {\left(\frac{C_{1}+C_{2}}{2}-H_{0}\right)}^{2}+\bar{\kappa }C_{1}C_{2}.$$

Such an analytical form of bending energy is often called Helfrich curvature free energy. *κ* 0 is the bending modulus of the film and $\bar{\kappa }$ is the saddle splay modulus that rules the preferred interface topology. If a disconnected structure is favored (w/o and o/w), $\bar{\kappa }$ 0 so that the formation of a spherically bent film with *c*_1_ and *c*_2_ having equal sign decreases the free energy. If bicontinuous μEs are favored, $\bar{\kappa }$ 0, because, in this case, the formation of saddle-shaped interfaces having an opposite sign for *c*_1_ and *c*_2_ decreases *g*. As a sign convention, it is customary to adopt the curvatures toward oil (o/w) as positive.

The total curvature free energy density, *G_c_*, for a given surface configuration is then formally obtained by integrating *g* over the specific area ∑

(20) $$G_{c}=\int_{\sum }gdA=\int_{\sum }2\kappa {\left(H-H_{0}\right)}^{2}dA+\int_{\sum }\bar{\kappa }KdA.$$

A feature of Equation (20) is that the integral of the Gaussian curvature term is constant with respect to changes in size and shape unless the film changes its topology (i.e., the film breaks). This is the consequence of the Gauss-Bonnet theorem of differential geometry [11] and it is the reason why, in most of the cases, one cares mainly about the term depending on the spontaneous curvature *H*_0_.

When the spontaneous curvature is null (*H*_0_ = 0) the curvature energy is minimum for *H* = 0 and the formation of bicontinuous μE and/or lamellar phases is favored.

In the case of C*_N_*E*_J_* nonionic surfactants, the ethoxy-chains are swelled by water at low *T* but at high temperatures, water behaves like a bad solvent. The result is a strong T-dependence of *H*_0_ that changes sign at the balance temperature *T*_0_ according to [52,73]:

(21) $$H_{0}\left(T\right)=\alpha \left(T_{0}-T\right)+\ldots$$

where *α* ≈ 10^−3^ Å^−1^ K^−1^. The symmetric shape of the μE domains in the χ-plot (see Figure 8A) reflects the fact that the free energy is invariant upon exchanging water and oil domains (changing *ϕ_o_* to *ϕ_w_*) and at the same time reversing the sign of *H*_0_ by crossing the balance temperature.

More in general the total free energy *G_tot_* of a μE is given by the sum of the curvature energy *G_c_*, the interfacial free energy $G_{i}=\int_{\sum }\gamma dA$ and an entropic term that in the case the μE is made by droplets can be written as *n_mic_k_b_Tf*(*ϕ*). The interfacial tension in μE is extremely low so that the leading terms are the entropic term and the curvature energy. For spherical droplets of radius *R_s_* Equation (20) gives [74]

(22) $$\frac{G_{tot}}{A}=\gamma +2\kappa {\left(\frac{1}{R_{s}}-H_{0}\right)}^{2}+\frac{\bar{\kappa }}{R_{s}^{2}}+\frac{k_{b}T}{4\pi R_{s}^{2}}f\left(\phi \right).$$

where *A* is the total interfacial area and *f(ϕ)* is a function of the dispersed phase + surfactant volume fraction *ϕ*. For dilute μEs, *f(ϕ)* reduces to *f(ϕ)* ≈ ln(*ϕ*) − 1. The different expressions for the mixing entropy of microemulsions can be found in Appendix B of a paper by Gradzielski et al. [74].

Under the condition of spherical droplets, *G_c_* has a minimum for an optimal radius *R*_0_ given by [75]:

(23) $$R_{0}=H_{0}^{-1}\left[\left(1+\frac{\bar{\kappa }}{2\kappa }\right)+\frac{k_{b}T}{8\pi }f\left(\phi \right)\right]$$

Any tentative to obtain droplets with size larger than *R*_0_ by adding dispersed phase results in the emulsification failure. Note that in these cases (*H*_0_ ≠ 0) the optimal curvature (1/*R*_0_) does not coincide with *H*_0_ [72]. Equation (23) allows the evaluation of the phase boundaries in the case of Winsor I and II phase separations. According to Equation (1), the optimal radius corresponds to an optimal ratio between the dispersed phase and the surfactant. When the entropic term can be neglected, the emulsification failure boundary, in a triangular phase diagram, is a straight line that originates from the solvent corner and cuts the dispersed phase-surfactant axis at the optimal ratio. Since *f(ϕ)* is always less than zero and becomes more and more negative with the dilution, the optimal radius increases with the surfactant concentration.

The case of the bicontinuous microemulsion is much more complicated. The quadratic expansion for the curvature energy does not rationalize the experimental phase behavior. Going beyond the harmonic approximation one finds [76,77]:

(24) $$G_{c}/V=a_{3}\phi_{s}^{3}+a_{5}\phi_{s}^{5}.$$

where *a*_3_ and *a*_5_ depend on *H*_0_, *κ* and $\bar{\kappa }$ but not on the volume fraction. According to the experimental phase diagrams, *a*_3_ 0 and *a*_5_ 0 for the μE while *a*_3_ 0 for the lamellar phase.

Accordingly, knowing the values *H*_0_, *κ* and $\bar{\kappa }$ it is possible to predict the whole behavior of the μE [78]. The complexity of the approach depends on the μE microstructure. In the case of disconnected o/w and w/o it is relatively simple to apply Equations (1) and (23) to predict the maximum solubilization before the emulsification failure and the shape transition from cylinder to the sphere above a critical *ϕ/ϕ*_s_ ratio [51]. Furthermore, the explicit presence of a contribution of saddle-shaped interfaces to the free energy ($\bar{\kappa }K$) allows the straight management of living networks [51].

On the other hand, the quantitative prediction of the phase behavior of bicontinuous and sponge μEs (e.g., the maximum swelling by oil and water) through the physics behind Equation (24) is, honestly, cumbersome. Furthermore, the experimental evaluation of *H*_0_, *κ* and $\bar{\kappa }$ is not trivial. Typically, both modules are of the order of few *k_B_T* so that the interface is flexible but the cost to have *H* ≠ *H*_0_ is substantial. Their determination requires sophisticated SANS and ultralow interfacial tension measurements [74]. Furthermore, the pieces of information obtained for a system cannot be translated to another one: any change in *T*, salinity, oil, and surfactant composition corresponds to a different set of *H*_0_, *κ* and $\bar{\kappa }$.

### 5.5. Hydrophilic–Lipophilic Difference (HLD)

A large part of the μE phase behavior can be understood simply in terms of how the intensive variables such as temperature and concentration of some components (electrolytes and alcohols mainly) impact the spontaneous curvature of the interfacial film. In the seventies, during investigations on the surfactant used in enhanced oil recovery (EOR), the balanced μEs found in a Winsor III phase separation were associated with an equal chemical potential of the surfactant in the excess aqueous ($\mu_{s}^{w}$) and oil ($\mu_{s}^{w}$) phases and, in general, their difference, normalized by the thermal energy, is called the hydrophilic–lipophilic difference (*HLD*) [79,80]:

(25) $$HLD=\frac{\mu_{s}^{w}-\mu_{s}^{o}}{RT}.$$

The chemical potentials can be split into additive contributions accounting for the effect of the oil and surfactant nature, temperature, ionic strength, and so on. Accordingly, a first set of empirical correlation equations was proposed to find the balanced (*HLD* = 0) condition [54,80,81]:

(26a) $$\begin{array}{cc} For\;anionic\;surfactant: & HLD=Cc-kEACN-c\left(T-25{}^{\circ}C\right)+lnS \end{array}$$

(26b) $$\begin{array}{cc} For\;nonionic\;surfactant: & HLD=Cc-kEACN+c^{\prime }\left(T-25{}^{\circ}C\right)+bS \end{array}$$

These equations are found to be robust for μE systems. In Equation (26a,b) the nature of surfactant and oil are characterized by the numerical values of the term *Cc* and *EACN*, respectively. The *EACN* is the equivalent alkane carbon number of the oil that, in the case of linear alkanes, corresponds to the number of carbon atoms. In all the other cases, *EACN* must be determined experimentally. The constant *k* ~ 0.15 suitably scales the *EACN* for a fair sum with other terms. The influence of ionic strength enters through the salinity (*S*) of the system expressed as the grams of NaCl in 100 mL of water. The logarithmic relation between salinity for ionic surfactants is consistent with the fact that in these kinds of systems, a zero salinity may not occur since the surfactant itself influences the ionic strength. For this reason, when the NaCl concentration in water is less than 1% in weight, correction is required considering the non-negligible surfactant concentration. Otherwise, the salinity contributes linearly for nonionics which are less sensitive to change in the ionic strength. The opposite sign in the correction for the temperature in the case of ionic and nonionic surfactants accounts for opposite morphology of the μEs observed when crossing the balance temperature; *c*~0.01 °C^−1^ *c’*~0.06 °C^−1^.

Lately, the term *HLD* has been associated with the spontaneous curvature of the interfacial film normalized by its thickness [82,83]:

(27) $$HLD∼-H_{0}l.$$

According to Equation (27), a positive *HLD* corresponds to *H*_0_ 0 (w/o), a negative *HLD* corresponds to *H*_0_ 0 (o/w) and for null *HLD* the μE is balanced (*H*_0_ ≈ 0). Accordingly, the term accounting for the surfactant hydrophobicity was called the surfactant characteristic curvature *Cc*. A wide database of *Cc* and *EACN* values are listed in many works by Salager, Acosta, Aubry, and Harwell [79,82,84,85,86,87,88] allowing the prediction of the *HLD* (*H*_0_) of several μEs.

If at a given: temperature, oil and ionic surfactant the μE is balanced by a salinity *S* = S*, then it is possible to predict the evolution of the film spontaneous curvature upon changing the salinity according to:

(28) $$HLD=-H_{0}l=ln\left(\frac{S}{S^{*}}\right).$$

**Figure 12:**
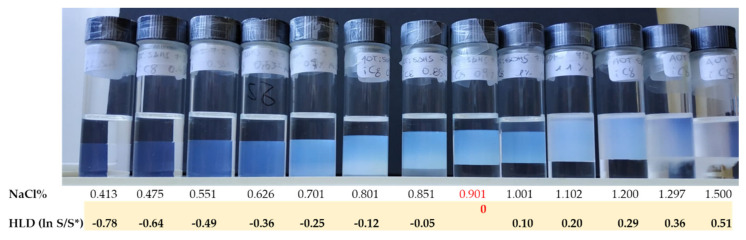
Example of salinity scan for a mix of sulfosuccinate surfactants in isooctane and brine.

An example of a salinity scan is shown in Figure 12. Such an approach also holds for mixtures of oils and surfactants of unknown *EACN* and *Cc* if their composition does not change during the salinity scan.

The additivity of the terms in Equation (26) makes it possible to determine the *Cc* or the *EACN* for a surfactant or oil, respectively, that are not yet in a database. The procedure consists of finding the optimal salinity for a μE made by the unknown surfactant (or oil) and oil (or surfactant) for which the *EACN* (*Cc*) is known. This is extremely useful for real-life applications employing a mixture of oils of natural or petrochemical origin. Another aspect that has made the *HLD* very popular is that, in many situations, the *Cc* of a mixture of surfactants is the mol-weighted average of their individual *Cc*s and the *EACN* of an oil mixture is the weight-weighted average of their individual *EACN*s. Of course, for situations where there are strong specific interactions as between cationic and anionic surfactants, such a rule cannot hold. At the basic level, the *HLD* is a shortcut to gain insight into the film spontaneous curvature (*H*_0_) or equivalently on the spontaneous effective packing parameter (*PP*°) that can be successfully used in a large number of practical applications [54,89].

The knowledge of the *HLD* parameters entering Equation (26) allows us to properly tune the film curvature by changing temperature or composition. This lets to design processes leading to the formation of a kinetically stable emulsion having small droplets (nanoemulsion). The strategy is to start from a balanced Winsor III equilibrium where, due to the ultralow surface tension, a gentle stirring is enough to emulsify the excess oil and water in form of very small emulsion droplets. The system is therefore abruptly moved away from such a state by a sudden change in one parameter such as the temperature or the addition of a different surfactant. Under the new conditions, the system is in a different Winsor equilibrium and at a higher interfacial tension that hinders the deformation of the interfacial film unavoidably associated with the coalescence of droplets made by the excess component (oil or water depending on the Winsor equilibrium).

### 5.6. Net Average Curvature (NAC)

Despite its potential ease of use, the *HLD* is a quantification of the preferred (spontaneous) state of the “unfrustrated” film. Indeed, it depends only on the temperature and the nature of the oil, surfactant and brine and not on their quantity. What is missing is the role of the actual curvature that is imposed by the compositional constraints (*ϕ_o_*, *ϕ_w_*, *ϕ_s_*).

In the case of the emulsification failure boundary observed for disconnected droplets, the model could be implemented expressing *H*_0_ in Equation (23) in terms of *HLD* using Equation (27) and neglecting the elastic and entropic contribution. Experimentally, such an assumption falls down when *HLD* approaches the balanced condition that is a super solubilization region where samples appear bluish, and the μE are bicontinuous [90]. This is also the situation where the application of the flexible surface model becomes cumbersome.

The Net Average Curvature (*NAC*) model is thus introduced to improve correlations and predictions in the transition regime. A comprehensive review of the *NAC* model has been published a few months ago by one of the proponents of the model [89], so that here we limit ourselves to a brief summary. The mathematical approximation of two coexisting states was exploited to handle the complexity of bicontinuous μE microstructure. One state is made by o/w with radius *R_o_*, and the other is made by w/o droplets with radius *R_w_*. With respect to these two fictitious states the Net Curvature equation (Equation (29)),

(29) $$H_{n}=\frac{1}{R_{o}}-\frac{1}{R_{w}}=-\frac{HLD}{l}$$

and the Average Curvature equation (Equation (30)) are introduced.

(30) $$H_{av}=\frac{1}{2}\left(\frac{1}{R_{o}}+\frac{1}{R_{w}}\right)\geq \frac{1}{\xi }$$

These are two new parameters defined to properly describe the μE. The sign of the net curvature *H_n_* is dictated by the *HLD* according to Equation (29). For a given system of oil, surfactant, and brine at a certain temperature, the *HLD* is calculated according to Equation (26). If the *HLD* is positive, the net curvature is negative meaning that a w/o μE is favored. In such a case, there is no oil droplet in the real system because the oil is the continuous phase. However, the NAC recipe requires the evaluation of the radius of the hypothetical droplet containing all the oil within the envelope made by the available surfactant. This can be calculated according to Equation (1) written for o/w droplets as $R_{o}=\frac{3\phi_{o}l_{s}}{\phi_{s}}=\frac{3\phi_{o}v_{s}}{\alpha \phi_{s}}$ (in the last equality, *v_s_ = αl* is the molecular surfactant volume). The corresponding radius of the w/o reverse micelles is given by:

(31) $$\frac{1}{R_{w}}=\frac{HLD}{l}+\frac{\alpha \phi_{s}}{3\phi_{o}v_{s}}.$$

For o/w droplets (*HLD* 0), an analogous equation holds: $\frac{1}{R_{o}}=\frac{HLD}{l}-\frac{\alpha \phi_{s}}{3\phi_{w}v_{s}}$. Note that in the conditions under which the oil is the continuous phase (*ϕ_o_* *ϕ_s_*), the *R_o_* is very large and its reciprocal is usually negligible in Equation (29). However, when the conditions are such that *HLD* ≈ 0 ≈ *H_n_*, both the radii in Equation (29) tend to diverge. To avoid this unphysical result, the constraint of Equation (30) is introduced: what is called the average curvature cannot exceed the interfacial film persistence length ξ. The persistence length can be achieved by small-angle scattering experiments or, much more easily, by the *ϕ_o_*, *ϕ_w_* and *ϕ_s_* within the bicontinuous μE according to Equation (3). Imposing for bicontinuous μEs that *H_av_* = 1/*ξ* the phase volumes and solubilization curves built according to the *NAC* approach reproduce the experimental data for several systems [89,90]. The predictive power of such a model has been widened by semiempirical relations linking surfactant structure to the parameters *l* and *ξ* entering Equations (29) and (30). Any estimate for the aggregate size *R* can be translated into a rough estimate for the interfacial tension of a thermodynamically stable μE and the liquids in equilibrium with it: the energy and the area involved are the thermal energy and the aggregate interface so that γ must be of the order of few *k_b_T/*4*πR*^2^. In turn, R and γ can be combined to predict a number of properties ranging from the emulsion stability to μE viscosity and wettability (for a recent review, see [89]).

## 6. Achievements and Challenges

### 6.1. Modern Applications

The applications of µEs include a wide span of areas such as food science, detergents, lubricants, coatings [91], antibacterial and pesticides, cosmetics [92,93], drug delivery, nanoparticle synthesis, biotechnology, isolation and extraction, chemical reactors, templates for gelification and even works of art restoration [94]. Here, we provide a brief description of the latest applications of µEs in some selected areas:

#### 6.1.1. Reaction Media for Synthesis and Catalysis

µEs have an extremely large and dynamic interfacial area which can disintegrate and reform in the millisecond scale [95]. This characteristic structure favors the kinetics of reactions taking place in the nanometer-sized droplets and allows their application as microreactors in several catalytic processes [96,97,98,99]. In this respect, one of the main characteristics of µEs is the possibility of coexistence of polar and nonpolar solvents. A good µE can, therefore, dissolve reagents of very different polarities which can be in contact due to the very large interfacial areas. The advantages in the use of μE’s in organic synthesis have been summarized in a review by Holmberg, where challenging aspects such as reagent incompatibility, slow reaction rate, and regioselectivity are discussed [100]. One of the earliest examples of the use of µEs to solve the issue of reagent incompatibility was presented by Menger and Elrington, when in 1991, they explored the use of µEs as media for the oxidation of half-mustard [101]. The addition of water-insoluble half-mustard to an o/w µE results in its solubilization in the droplets. Further addition of hypochlorous acid (OHCl) leads to oxidation of the half-mustard to a sulfoxide via the formation of butyl hypochlorite at the droplet–water interface. Gutfelt et al. demonstrated the convenience of using µEs over two-phase systems with phase transfer reagents in the synthesis of decyl sulfonate in 1997 [102]. The surfactant was formed through a nucleophilic substitution reaction from decyl bromide and sodium sulfite. The reaction’s rate was found to be faster in the µE while it ceased in the two-phase system.

The tiny droplets of µEs have also been widely used as microreactors for the synthesis of organic and inorganic nanoparticles [4,102,103]. Inorganic nanoparticles have been customarily synthesized by w/o µEs. The strategy consists of mixing a µE containing the precipitating agent with another one containing the metallic precursor [104]. The droplets will collide and coalesce upon mixing, producing reactant interchange and leading to nucleation and growth of the nanoparticles which will be confined in the µE’s droplets. Another approach consists of preparing a µE with one reactant and adding the other one as a solution. Alternatively, all the reactants can be dissolved in the polar phase of the µE and the nucleation is then induced via changing operative parameters such as pH, temperature, etc. Finally, there is a fourth approach that relies on the addition of one reactant in the polar phase and the other in the apolar phase, so that the reaction is limited to the liquid–liquid interface. This latter approach is specifically effective in the synthesis of hollow nanospheres [105]. The literature on this topic has grown steadily during the last 20 years, producing many articles and reviews about size and shape control, synthesis mechanisms, etc. [106,107,108,109,110,111,112,113,114,115]. Some examples of the great variety of nanoparticles produced using µEs include metallic and bimetallic nanoparticles (Au, Ag, Pt, Pd, Cu, etc. Pt/Pd, Ag/Au, Ag/Cu), single and mixed metal oxides, quantum dots, semiconductors, polymers, and biomacromolecules [106,107,116,117,118,119]. On the other hand, the use of enzymes as biocatalysts for large-scale industrial processes can be challenging since enzymes need a very precise aqueous environment with a correct pH and temperature to function. Changing these parameters or a too hydrophobic medium could lead to enzyme denaturation. However, some of the most important chemical reactions use hydrophobic substrates. Therefore, the use of w/o and bicontinuous-μE has been a very important approach in the last two decades [120,121,122,123,124]. In this approach, the enzyme and the hydrophobic reactant are solubilized in the aqueous phase and the organic solvent, respectively, and the reaction takes place at the interface between both phases. Using this method the enzymes are trapped in tiny water domains, being protected from undesired contact with the surrounding polar medium while maintaining their activity [125].

A series of studies performed during the last decade has proved the replacement of conventional organic solvents by Ionic Liquids (ILs) or CO_2_ to be very advantageous [5,126,127,128,129,130,131]. ILs have many attractive and unique properties such as negligible vapor pressure, high conductivity, thermal stability, broad liquid range, and good tunability by using cation-anion combinations [132]. They are, therefore, a good alternative to organic solvents, given their much lower environmental impact [133]. In the field of organic synthesis, many reactions need to be performed in a water-free environment. In this regard, ILs-µEs using nonionic surfactants are an interesting reaction medium since ILs can be chosen to be either hydrophobic or hydrophilic [134]. A kinetic study performed by Matalobos et al. found the rate constant for the aminolysis of nitrophenyl laureate to be 2–4 times higher in IL/O µEs ([bmin][BF_4_]/TX-100/cyclohexane) than in w/o µEs [135].

#### 6.1.2. Nanoparticle Synthesis

Much work has been done in recent years in the synthesis of nanoparticles using ILs-based µEs. The group of Serrà et al. has proposed the use of [bmin][PF_6_]/H_2_O/TX-100 to prepare mesoporous CoPt [136] and CoNi@Pt [137] for alcohol fuel cell applications. The nanopores distribution was successfully tuned using diverse µE systems allowing to obtain nanorods with very high surface areas and excellent corrosion stability. The same group succeeded in producing mesoporous nanorods with CoNi@Au core–shell for magnetic drug nanocarriers applications using ionic liquids-in-aqueous µEs [138]. Due to the high effective area and magnetic character of the nanocarriers, the needed amount of both drug and nanocarrier in the treatment of cancer can be dramatically reduced.

Pei et al. proposed a novel high-temperature µE system consisting of IL mixtures designed as a microreactor for high-temperature reactions. The µE droplets were demonstrated to be stable up to 200 °C, and the size distribution tuned by varying the composition and selection of ILs of the system [139].

#### 6.1.3. Drug Delivery

µEs are of great importance to the pharmaceutical industry since they are excellent candidates for drug delivery systems. Some of the advantageous characteristics of µEs include improved drug solubilization, long-term stability, high encapsulation efficiency, biocompatibility, and ease of preparation and administration [140]. Furthermore, µEs play important roles in the sustained release of local, ocular, oral, nasal, transdermal, and parenteral drug delivery [141,142,143,144]. Additionally, w/o µEs are particularly interesting for oral protein delivery due to their increased oral bioavailability through the gastrointestinal tract and their protection capacity [144]. A large number of drugs, such as antibiotics [141,142,143,144,145,146], antifungals [147,148,149], anti-inflammatory [150,151,152,153,154] and immunosuppressive [155] molecules have been used in μEs systems.

An example of such applications is the development of insulin-loaded chitosan nanoparticles trapped in a w/o µE formed by water (PBS solution), oil (Plurol Oleique), surfactant (lecithin and Tween 80), and a cosurfactant (ethanol). In vitro studies showed insulin retained its structure after encapsulation and a retarded release under simulated gastric conditions. On the other hand, in vivo studies in rats showed this system succeeded in controlling blood glucose levels after oral administration [144].

Among many other properties, curcumin interferes with the amyloidogenesis and inhibits the Aβ aggregation in the brain [156]. In 2012, Wang et al. developed a µE-based in situ ion responsive gelling system loaded with curcumin for brain delivery by intranasal administration [157]. In vitro studies revealed a sustained release of the drug up to 24 h and in vivo investigations showed no damage to the nasal mucosa and a significant increase in the brain uptake of the drug.

One of the possible disadvantages of aqueous µEs for drug delivery systems is their limitation to water-soluble drugs. This limitation can be overcome by the use of alternative solvents such as ILs [158]. Using such an approach, Goindi et al. reported the synthesis of an IL/w µE for the topic delivery of a poorly water-soluble drug, etodolac, which is used to treat arthritic symptoms [42]. The IL-based µE showed a 99% permeation efficiency compared to the o/w μE (61%) and the oil solution (49%). Histopathological studies showed the IL-based µEs inferred no anatomical or pathological changes in the skin, thus implying the safety of the drug delivery system.

Another example of the use of ILs for enhanced drug delivery was recently presented by Wang et al., who reported an imidazolium IL-based µE for the topical delivery of Dencichine, which is a promising drug for the treatment of hemorrhage. The numerous side effects provoked by the oral administration of Dencichine motivated the authors to find an alternative transdermal route of administration. Dencichine is water-soluble, which is related to low efficiency in transdermal delivery. However, the incorporation of the drug in the imidazolium IL-based µE allowed for a 10-fold increase in topical permeation efficiency compared to conventional aqueous drug solutions [159].

#### 6.1.4. Pesticides and Antibacterial

µEs have been suggested to possess antimicrobial properties due to the impossibility of bacteria to survive in pure oil. Furthermore, the observed antimicrobial activity of µEs has been related to their direct effect on the bacterial cytoplasmic membrane, resulting in cell wall modifications, cytoplasmic coagulation, disruption of intracellular metabolism, and cell death [160]. Furthermore, conventional formulations of pesticides are based on petroleum and organic solvents, which are being gradually replaced by water-based formulations such as µEs [161].

Teixeira et al. studied the use of an o/w µE (ethyl oleate/Tween 80/water with n-pentanol as cosurfactant) to act against suspensions of vegetative cells and preformed biofilms of *Salmonella typhimurium*, *Escherichia coli*, *Pseudomonas aeruginosa*, *Staphylococcus aureus*, and *Listeria monocytogenes*. The µE effectively attacked all five organisms, both as suspensions or formed biofilms [162].

Hu et al. have proposed the formulation of a µE for the application of antimicrobial SecA inhibitors as a treatment for citrus disease, Huanglongbing (or citrus greening). The prohibition of antibiotics such as streptomycin or tetracycline in the use of citrus greening motivated the authors to look for an alternative antibacterial treatment. SecA is an essential protein in all bacteria and is a promising target for antibacterials and its inhibitors are poorly water-soluble. The development of a µE (consisting of N-methyl-2-pyrrolidone and dimethyl sulfoxide as solvent and cosolvent, as well as polyoxyethylated castor oil, polyalkylene glycol, and polyoxyethylene tridecyl ether phosphate as surfactants) significantly increased SecA inhibitors, which proved to have antibacterial activities comparable to that of streptomycin [141].

Another type of µE formulation was recently proposed by Shao et al. to control diamondback moths (*Plutella xylostella*). The prepared µEs were composed of norcantharidin/Tx13 and Tw80/water and ethanol as a cosurfactant. The insecticidal bioassay indicated the acute LC50 to *P. xylostella* to be 12.5 mg/mL, which implies a promising environment-friendly pesticide alternative.

#### 6.1.5. Food Technology

The main implication of amphiphiles in the food sector deals with emulsion manufacturing like in the case of dairy products, creams, soft drinks, etc., obtained through energy-demanding processes.

On the contrary, the exploitation of thermodynamically stable microemulsions’ properties in food technology has always been considered more challenging.

The main applications of food microemulsions can be summarized in (i) nutraceutical delivering/protective system [163,164,165,166,167] and (ii) food manufacturing and sanitization [167,168,169].

Often nutraceutical compounds are poorly water-soluble molecules like vitamins, essential fatty acids or, more in general, phytochemicals and a promising application is to obtain functional foods by either improving their solubility in water-based matrices or protecting them by unfavorable events like oxidative damage, proteolytic damage. A suggestive recent application in these terms is proposed by Xu et al. in 2016 and deals with the preparation and characterization of different cosurfactant free o/w microemulsions using Tween 80 as a surfactant and microalgal oil as the organic dispersed phase. The microalgal oil used in the work is reported to be composed of considerable amounts of docosahexaenoic acid (DHA), up to 41.3%, that is easily oxidized if exposed to light, high temperatures, and oxygen. The authors reported the stability of the system after 24 months of investigation monitoring peroxide values and particle size changes after UHT processes and CaCl_2_ addictions [166].

Concerning food manufacturing, Amiri-Rigi and Abbasi reported the application of different lecithin-based olive oil microemulsions as extracting solvent for lycopene from tomato pomace. The lecithin is used together with 1-propanol in a constant mass ratio of lecithin: 1-propanol of 2:1 in all the systems under investigation. A ternary phase diagram is reported, and the extraction efficiency is tested for 10 samples taken from two dilution lines. Four extraction cycles made using the microemulsion composed of lecithin/1-propanol:olive oil:deionized water in a mass ratio of 80:10:10 led to an extraction efficiency of 88% [167]. An example of the application of microemulsions as washing solutions for fruit and vegetables is provided by Zhang et al. with the purpose of potentially replacing sanitizers commonly used to prolong shelf-life of products and enhance safety during manufacturing steps. Different o/w microemulsions composed by a mixture of lecithin and a commercial product based on sucrose octanoate ester (SOE) (40% *w*/*w*) have been prepared by adding tunable amounts of essential oils. The essential oils investigated are the clove bud oil, the cinnamon bark oil, and the thyme oil and are potential antimicrobial agents. The study is mainly focused on microemulsion stability and direct contact angle measurements on fresh produce [169].

### 6.2. The Challenge of Biosurfactants

Biosurfactants (or Bios) represent a new class of sustainable surfactants which have kept the interest of scientists in the last decades (for a very recent review on this topic, see [170]). Researchers and industries took an interest in biobased surfactants at the start of the 2000s because of the variation in crude oil prices and the following the rise of fuel-based surfactants price. However, when in 2009 oil prices stabilized the focus on biosurfactants stayed on. Currently, fossil fuel-derived surfactants are less expansive, but this trend will, likely, reverse in the future. Moreover, during these years of research, many interesting properties of these green surfactants have come out. Indeed, these biobased molecules have interfacial activity [171,172,173,174,175,176,177,178,179,180,181,182] as their petroleum-based counterparts but also show remarkable bioactivity [183].

There is no unambiguous definition of biosurfactant: this term describes at the same time amphiphilic secondary metabolites secreted by microorganisms, biodegradable surfactants, and surfactants produced from renewable sources [184]. However, most of the time, biosurfactants are lipids, proteins, and sugars that build the cell wall of bacteria, yeasts, or fungi [185]. These surface-active agents are synthesized by microorganisms in order to provide several advantages in an ecological niche [186]: they have antibacterial and antimycotic activity against enemies, they might enhance the bioavailability of hydrophobic nutrients and ensure attachment to surfaces forming biofilms. Comprehensive tables of biosurfactants are collected elsewhere [184,185,187].

Biosurfactants may be classified in several ways according to their charge, size, or structure [184]. Bios can be anionic, cationic, or nonionic as other common surfactants but there are also amino acid-based surfactants or amphoteric biobased surfactants such as betaines containing both negatively and positively charged groups. According to their structures, Bios can be divided into glycolipids, lipopeptides, and proteins; glycolipids are the most important molecules commercially available as future competitors of ethoxylate-based surfactants [188,189]. An immediate method of classification allows us to split them all into two groups as low-molecular-weight and high-molecular-weight biosurfactants. Lately, a classification based on their renewable-carbon content was proposed by CEN [190].

Nowadays, biobased surfactants are suitable for many tasks, but only cosmetic, food, and pharmaceutical industries could absorb the higher cost of Bios [191]. Researchers have spent significant effort in the development of practical approaches to make biosurfactants economically attractive in a large-scale production [187,192]: using cheap raw materials, optimizing fermentation and purification processes, and developing overproducing nonpathogenic mutants.

In most of their applications, microbial surfactants substitute their synthetic counterparts. They are suitable for EOR [193,194] and nanoparticle synthesis [195,196,197] replacing more environmentally hazardous chemicals. On the other hand, their effects on human and animal cells open many doors in new fields of application [183]. In medical applications, they have been used as emulsifying aids for drug delivery, as a supplementary pulmonary surfactant, as adjuvant of vaccines and as antiadhesive coating agents for medical insertional materials [198]. In environmental applications, the use of microbial surfactants has been proposed for bioremediation of soil, water, and waste [199]. In cosmetic formulations, they can replace nonbiodegradable surfactants and can also play a role as prebiotics [200] while in the food technology they are exploited nonfouling agents avoiding the binding of microbes [201].

One of the main issues with biosurfactants is the unpredictable composition of their ingredients: often a type of Bios includes more than one single molecular structure. Biobased surfactants are produced as a mixture of different congeners with different chain length, saturation, and functional groups. Therefore, many classification methods are required, as previously described.

In order to overcome this issue a rapid and cheap method to assess the biobased formulation conditions is the most practical way for Bios to become truly viable. The *HLD* model seems the best opportunity to achieve this goal. As previously described in this review, the mixing rule is a robust tool useful in the case of surfactant mixtures. Characteristic Curvature values of the main available Bios are measured by many authors according to the *HLD*-*NAC* model and are reported elsewhere [84,202,203,204,205,206,207,208,209]. These *Cc* values are good starting points for fast quality control on Bios, then thanks to the mixing rule it is possible to quantitatively adjust *Cc* value by adding other surfactants [203].

Biobased surfactants seem a sustainable alternative to many essential chemicals in several fields of research and industries. In order to manage these new green materials a good knowledge of rational formulation models is needed, *HLD* is the most efficient method for managing Bios among all the techniques reviewed by the authors. A switch from fossil fuel-based to biobased surfactant is inevitable in a long way run to a responsible economy focused more on the environment and society.

## 7. Concluding Remarks

Over the last decades, microemulsions have been, and still are, very important and promising areas of study for a wide range of applications, from food science, detergents, and cosmetics, to drug delivery, nanoparticle synthesis, biotechnology, etc. This tutorial review summarizes the possible microstructures and phase behavior found in µEs, including the customary techniques and theoretical models used to describe and formulate them. Additionally, some examples of the recent progress in the applications in diverse fields have been presented.

Since microemulsions are thermodynamically stable, optically isotropic, and transparent, one needs to make use of different instrumental and experimental methods to gain insight on the microstructure. Some of the most common instrumentation described in this review are diffusion NMR, electrical conductivity, DLS, SAXS, SANS) an electron microscopy. However, the use of experimental approaches, such as creating small perturbations in the composition, can also be very helpful in the microstructure’s determination. We limited the discussion to a schematic representation of phase diagrams depending on the typically observed phenomena upon variation of temperature and composition.

The formulation of µEs requires a deep understanding of the physical chemistry and the available theoretical models to determine their microstructure, and how to impact the interfacial tension and the overall performance. We have presented a description of the most important theoretical models used nowadays to study µEs including HLB, *CPP*, the flexible surface model, *HLD*, and *NAC*.

The interest in the extent of applications of µEs has been extensively demonstrated over the last decades. Nevertheless, the increasing number of applications and their unquestionable complexity further adds to the need for carefully reviewing and developing the existing theory to fully exploit the potential possibilities of µEs.
